# Comparative prevalence of the mercury resistance gene *merA* in human feces, food, and environmental water from Japan, Vietnam, and Ghana

**DOI:** 10.1371/journal.pone.0357976

**Published:** 2026-09-08

**Authors:** Yen Hai Le, Judith Dzifa Azumah, Diep Thi Khong, Thang Nam Nguyen, Cornelia Appiah-Kwarteng, Kazuaki Matsui, Mayumi Yamamoto, Kaori Tanaka, Yoshimasa Yamamoto

**Affiliations:** 1 The United Graduate School of Drug Discovery and Medical Information Sciences, Gifu University, Gifu, Japan; 2 Center for Medical and Pharmaceutical Research and Service, Thai Binh University of Medicine and Pharmacy, Hung Yen, Vietnam; 3 School of Veterinary Medicine, University of Ghana, Accra, Ghana; 4 Department of Civil and Environmental Engineering, Kindai University, Higashi-Osaka, Japan; 5 Health Administration Center, Gifu University, Gifu, Japan; 6 Institute for Glyco-core Research, Gifu University, Gifu, Japan; University of Agriculture Faisalabad, PAKISTAN

## Abstract

In this study, we investigated the prevalence and abundance of the mercury resistance gene *merA* in human feces, retail chicken meat, and environmental water samples collected from Japan, Vietnam, and Ghana. A real-time PCR assay developed in this study demonstrated high specificity toward *merA* sequences from more than 12 bacterial species. Using this assay, *merA* was detected in 6.8% of human fecal samples in Japan (n = 29), in contrast to significantly higher rates observed in Vietnam (70.2%, n = 47) and Ghana (97.4%, n = 39). Similar geographic trends were evident in the chicken meat samples: 18.5% in Japan (n = 27), 66% in Vietnam (n = 91), and 90% in Ghana (n = 10). Environmental water samples showed a consistently high *merA* detection rate across all countries (75–100%, n = 21), with substantially higher gene copy numbers in Vietnam and Ghana than in Japan. *merA* was detected in some water samples, even when total mercury concentrations were below the detection limit, indicating that molecular detection may offer greater sensitivity than traditional physicochemical methods. Mercury-resistant bacteria were successfully isolated and cultured, and *Citrobacter freundii* was identified as the representative strain. Genomic analysis revealed that *merA* was located on an IncFIB plasmid, flanked by insertion sequences, suggesting its potential for horizontal gene transfer. These findings highlight *merA* as a promising biomarker for environmental mercury exposure and support the utility of fecal *merA* analysis as a proxy for assessing mercury-related public health risks.

## Introduction

Mercury (Hg) is a widespread environmental pollutant due to its ubiquitous presence in nature and continuous release into soil and water. Both anthropogenic activities, such as mining, and natural processes, including volcanic eruptions and wildfires, contribute to Hg contamination of the environment [1]. Small-scale gold mining is a major contributor to global Hg emissions, especially in African countries such as Ghana [2].

In contaminated environments, the predominant inorganic form of Hg is Hg(II), which is highly toxic because of its strong affinity for cellular thiol groups, leading to disruption of essential biological functions. The bacterial *merA* gene plays a key role in detoxifying Hg(II) by reducing it to the less toxic and volatile elemental Hg (Hg^0^). In contrast, methylmercury (MeHg) is the Hg species of greatest concern for human health because it readily bioaccumulates and biomagnifies through aquatic and terrestrial food webs. Consequently, humans, as top-level consumers, are particularly vulnerable to MeHg exposure through dietary intake [3–5]. Accordingly, systematic surveillance of Hg contamination across environmental and dietary sources is a critical component of public health protection. Importantly, the World Health Organization (WHO) sets a guideline value of 0.006 mg/L for total Hg in drinking water [6]. However, national standards in both Vietnam [7] and Ghana [8] impose a more stringent maximum permissible concentration of 0.001 mg/L for both drinking and surface water, above which Hg exposure is considered hazardous to human health.

Microbial communities play a crucial role in regulating the Hg cycle [9]. Hg-resistant bacteria are enriched in Hg-contaminated environments due to the strong selective pressure imposed by Hg(II) toxicity [10]. Approximately 1–10% of heterotrophic aerobic microbes from various environments possess *mer* systems that confer resistance to Hg by detoxifying Hg(II) [11].

The bacterial *mer* operon is a well-characterized genetic system involved in Hg detoxification. It includes genes responsible for Hg transport, regulation, and reduction. Within this operon, *merA* encodes mercuric reductase, which catalyzes the NADPH-dependent reduction of Hg(II) to Hg^0^, thereby decreasing intracellular Hg toxicity [5]. Some *mer* operon variants also contain *merB*, which enables the degradation of organomercury compounds such as MeHg. However, *merA*-mediated reduction of inorganic Hg(II) remains the core mechanism of bacterial Hg resistance [12,13]. Horizontal gene transfer (HGT) plays a significant role in the dissemination of Hg resistance genes in aquatic ecosystems. Previous studies have documented the transfer of *mer* operon via conjugative plasmids and transposon among diverse bacterial taxa in freshwater and marine environments [27], highlighting the potential for rapid spread of Hg resistance determinants in response to environmental selection pressure.

Hg contamination can be detected through direct physicochemical analysis or by ascertaining indirect biological indicators, such as the prevalence of Hg-resistant bacteria. The abundance of Hg resistant bacteria may reflect the increased presence of bioavailable Hg [14,15]. The quantification of *merA* in microbial communities, including human gut microbiota, food-contaminating bacteria, and environmental microorganisms, offers valuable insight into environmental Hg exposure and the associated potential health risks. Indeed, a study in an aquatic environment has successfully used *merA* expression, measured by mRNA production, as a direct indicator of bioavailable Hg [16].

In this study, we focused on the Hg resistance gene *merA* as a biomarker for regional environmental Hg contamination. We investigated the prevalence and abundance of *merA* in fecal samples from local residents, food, and environmental water across three countries (Japan, Vietnam, and Ghana). Furthermore, we evaluated its potential utility as an indicator of Hg contamination.

## Materials and methods

### Ethical approval

Clinical specimens were collected from study participants following the approval of the institutional ethics committees at Gifu University (no. 2019–164), Thai Binh University of Medicine and Pharmacy (no. 1264/HDDD), and the University of Ghana (no. ECBAS 051/22–23). Written informed consent was obtained from all participants and/or their legal guardians.

### Sample collection

This study utilized fecal samples provided by local residents, commercially available chicken meat, and environmental water obtained from public water bodies. The collection of small quantities of retail chicken meat and environmental water for research purposes is not subject to regulatory restrictions. The use of human fecal specimens was conducted with the approval of the ethics committee of the relevant local research institution.

This study was conducted using newly collected samples from Ghana and Vietnam in addition to previously reported samples from Vietnam and Japan. Details are provided in Tables 1–3.

**Table 1:** Characteristics of participants.

Country	City	Date of sampling	Participants	Median age (years)^b^	Age range (year)	Gender % male
Ghana	Kumasi	December, 2023	39	29	10-85	27.6
Vietnam	Thai Binh	March, 2024	47^a^	41	3-93	46
Japan	Gifu	April, 2024	29^a^	20	18-54	17

^a^, The samples used in this study were collected in a previous study by Yamamoto, et al., Microbiol Spectrum, e0292024, 2025.

^b^, The decimal points were rounded off.

**Table 2:** Characteristics of chicken meat samples.

Country	City	Date of sampling	Number of samples
Ghana	Kumasi	Feb. 2023	10
Vietnam	Ho Chi Minh	Jan. 2023	10^a^
	Nghe An	Jun. 2023	10^b^
	Hanoi	Sep. 2023	11^b^
	Thai Binh	Oct. 2023	12^b^
	Hanoi	Mar. 2024	48^b^
Japan	Gifu	May. 2021-Aug. 2024	27^b^

^a^The samples were collected in a previous study by Le, et al. (Foodborne Pathog Dis, 21:485, 2024)

^b^The samples were collected in a previous study by Le, et al. (Int J Food Microbiol, 430:111061, 2025)

**Table 3:** Characteristics of enviromental water samples.

Country	City	Sampling location	Date of sampling	Sample ID	Hg physicochemical measurement	Culture on Hg-LB agar
Ghana	Accra	Lagoon close to the coast	Mar. 2025	Gha25-001W	-	Yes
		Lake		Gha25-002W	-	-
		Wetland waterway		Gha25-003W	-	Yes
		Lagoon close to the river		Gha25-004W	-	Yes
	Kumasi	Canal-community A	Mar. 2025	Gha25-005W	-	-
		Canal-community B		Gha25-006W	-	Yes
		Canal-community C		Gha25-007W	-	-
		Gutter-community D		Gha25-008W	-	-
Vietnam	Thai Binh	Canal-community A	Feb. 2025	TB25-001W	Yes	Yes
		Pond in the city		TB25-002W	Yes	Yes
		Canal-community B		TB25-003W	Yes	Yes
		Paris river		TB25-004W	Yes	-
		Canal-community C		TB25-005W	Yes	-
		Bo river		TB25-006W	Yes	-
		Canal-community D		TB25-007W	Yes	-
		Kỳ Bá Lake		TB25-008W	Yes	-
		Canal-community E		TB25-009W	Yes	Yes
Japan	Gifu	Canal on the university campus	Feb. 2025	GF25-001W	Yes	-
		Canal in rice fields		GF25-002W	Yes	-
		River		GF25-003W	-	-
		Household tap water		GF25-004W	-	-

-, not tested.

### Human fecal samples

Human fecal samples were procured for the experiment in this study. A total of 47 participants were recruited from Thai Binh Province (Red River Delta), Vietnam; 29 participants from Gifu Prefecture (Central Japan), Japan; and 39 participants from Kumasi (the Kumasi Metropolitan Assembly in a rainforest region), Ghana. Characteristics of participants and sampling date are depicted in Table 1. One fecal sample was collected from each participant. All samples were collected as previously described [17].

### Food samples

Retail chicken meat was used as a food sample. A total of 91 chicken meat samples were collected from meat retailers in four regions of Vietnam (Hanoi, Thai Binh, Nghe An, and Ho Chi Minh City). Additionally, 10 samples were obtained from meat retailers in Kumasi, Ghana, and 27 samples were collected from meat retailers in Gifu City, Japan. All chicken meat examined was locally raised. In each case, a single sample was collected from each retailer. Details of meat sample collection have been described in our previous reports [18,19]. An overview of meat samples is provided in Table 2.

### Environmental water samples

Environmental water samples were collected from local water sources in the target regions. Approximately 50 mL of each water sample was collected in a sterile container at the sampling site. A total of 21 environmental water samples were assessed in this study.

The samples were initially centrifuged at 211 *× g* for 1 min to remove insoluble residues. The supernatant was then subjected to high-speed centrifugation at 15,000 *× g* for 5 min, and the resulting pellet was collected. The obtained pellet was used for DNA extraction and isolation of Hg-resistant bacteria. Table 3 provides the characteristics of the environmental water samples.

### Mercury concentrations

In accordance with the official analytical method published by the Ministry of the Environment, Japan [20], the total Hg levels in environmental water samples were measured using cold vapor atomic absorption spectrophotometry (CV-AAS). Hg concentrations were measured in 11 environmental water samples from Vietnam and Japan. The sampling locations are listed in Table 3.

For each sample (29–52 mL), the debris and suspended solids were first removed by centrifugation at 211 × *g* for 1 min. Hg in the water samples was then reduced to its vapor phase for quantification, following the official analytical method. The analysis was conducted by a commercial vendor (Tokai Technical Center, Nagoya, Japan).

### Detection of the mercury resistance gene (*merA*)

The *merA* gene was detected in DNA extracted from each sample using real-time PCR. Briefly, DNA was extracted from the sample pellets using the Kaneka Easy DNA Extraction Kit version 2 in accordance with the manufacturer’s instructions. Specifically, 1 g of fecal matter, 10 g of chicken meat, and 50 mL of environmental water were used for DNA extraction, respectively. DNA concentrations varied depending on the sample type and extraction efficiency, typically ranging from 10-50 ng/μL for fecal samples, 5–30 ng/μL for chicken meat samples, and 0.5-10 ng/μL for environmental water samples. Approximately 100 μL of DNA solution was obtained from each extraction. A portion of the extracted DNA was then subjected to multiplex real-time PCR using a TaqMan probe-based assay on the Mic qPCR Cycler (BioMolecular Systems, Queensland, Australia). The *merA*-specific primers and probe sequences, as well as the PCR conditions, are detailed in S1 and S2 Tables. The *merA*-specific primers and probe were designed using Geneious Prime software (Geneious, Boston, MA, USA). For the *merA*-positive control, genomic DNA was extracted from *Escherichia coli* strain 22–245 (DDBJ accession no. AP038811) using the NucleoSpin Microbial DNA Kit (Marchery-Nagel, Düren, Germany) according to the manufacturer’s instructions. The concentration of *merA* in the samples was preliminarily quantified and expressed as *E. coli* DNA-equivalent copy numbers using the copy number calculation tool [21]. The PCR Cq values below the detection range of the standard curve were calculated by extrapolating the curve.

### Isolation of mercury-resistant bacteria

Hg-resistant bacteria were isolated from sediments derived from environmental water samples through centrifugation, following the protocol described above. The sediment was spread onto Luria-Bertani (LB) agar supplemented with 60 μM HgCl_2_ and incubated at 30°C for 20 h. The HgCl_2_ concentration was selected based on preliminary culturing experiments and previous studies using micromolar HgCl_2_ concentrations as selective pressure for isolating Hg-resistant bacteria [22]. Colonies developed on the plates were subjected to further subculturing on LB agar to obtain the isolates. The presence of the *merA* gene in the isolated colonies was detected using real-time PCR, and the bacterial species was identified using Matrix Assisted Laser Desorption Ionization Time-of-Flight Mass Spectrometry (MALDI-TOF MS, Bruker Japan, Kanagawa, Japan).

### Genome analysis

Genomic DNA was extracted from the Hg-resistant bacterial isolate *Citrobacter freundii* TB25-009W-60–9, which was recovered from an environmental water sample collected in Vietnam during the selective isolation described above, using the NucleoBond HMW DNA kit (Macherey-Nagel, Germany) according to the manufacturer’s protocol. Whole-genome sequences were obtained using a NovaSeq X Plus (Illumina, CA, USA) and MinION Mk1C sequencer (Oxford Nanopore Technologies, London, UK). Short-read sequencing was performed using the NovaSeq X Plus high-throughput sequencing set by a commercial vendor (Genome-Lead, Takamatsu, Japan). For long-read sequencing, performed using MinION with an R10.4.1 flow cell, and the high molecular weight DNA of each isolate was barcoded using a Rapid Barcoding kit (Oxford Nanopore Technologies). The MinION library was prepared without fragmentation and cleaned using AMPure beads (Beckman Coulter, CA, USA). To obtain the complete genome of the isolate, a *de novo* hybrid assembly of both short and long reads was conducted using Unicycler 0.5.1, with default settings, and CLC Genomics Workbench 25.0.1.

The completely assembled sequences were annotated by uploading the FASTA files to the DNA Data Bank of Japan Fast Annotation and Submission Tool v.1.6.0. [23]. To investigate antimicrobial resistance genes, the assembled sequences were screened and confirmed using the ResFinder 4.7.0 database [24]. Plasmid replicons were detected using PlasmidFinder 2.1 [25]. The sequences were further analyzed and visualized in Genious Prime v.2025.0.3, which was used to examine the organization and structure of the *merA* gene and to support comparative genomic analysis.

### Statistical analysis

Statistical comparisons of variables among the three countries were conducted using the Chi-square test with StatFlex version 7 (Artech Co., Ltd., Osaka, Japan). When expected samples in category were less than 5, Fisher’s exact test was applied. A *p*-value of <0.05 was considered indicative of statistical significance.

## Results

### Specificity of *merA* detection primers and probe

The specificity of the *merA* primers and probe used in this study was evaluated using BLAST analysis against genome sequences registered in GenBank. The results showed 100% identity with *merA* sequences from over 12 bacterial species (S3 Table), including *Escherichia*, *Morganella*, *Citrobacter*, and *Pseudomonas*.

### Validation of the method for *merA* detection

Using DNA from the *merA*-standard strain, a detection sensitivity of 0.3 pg DNA per reaction was achieved using the method (S1Fig). The quantifiable DNA range was 0.3 pg to 3 ng per reaction, corresponding to approximately 10⁴ to 10⁸ copies of *merA*.

### Prevalence of the *merA* gene in collected samples

The prevalence of the *merA* gene in human fecal samples from residents of multiple countries was assessed using *merA*-specific real-time PCR. The results are summarized in Table 4. In Japan, only 6.8% of the individuals tested positive for *merA*, whereas the prevalence was significantly higher in Vietnam (70.2%) and Ghana (97.4%) (*p* < 0.001).

**Table 4:** Prevalence of *merA* in human stools, food and water.

Country	Human stool		Chicken meat		Environment water	
	Positive/No. of sample tested	% positive	Positive/No. of sample tested	% positive	Positive/No. of sample tested	% positive
Ghana	38/39	97.4	9/10	90	8/8	100
Vietnam	33/47	70.2	51/91	66	7/9	77.7
Japan	2/29	6.8	5/27	18.5	3/4	75

A similar detection assay conducted on retail chicken meat samples revealed *merA* in 18.5% of samples in Japan, compared to 66% in Vietnam and 90% in Ghana (*p* < 0.001).

In contrast to the variation observed in human and chicken samples, environmental water samples (mainly from small local canals) collected from the same regions exhibited consistently high *merA* detection rates across all countries, ranging from 75% to 100%, with no significant differences (*p* = 0.337), as shown in Table 4.

### Abundance of *merA* across environmental, food, and human samples

**Fig 1:**
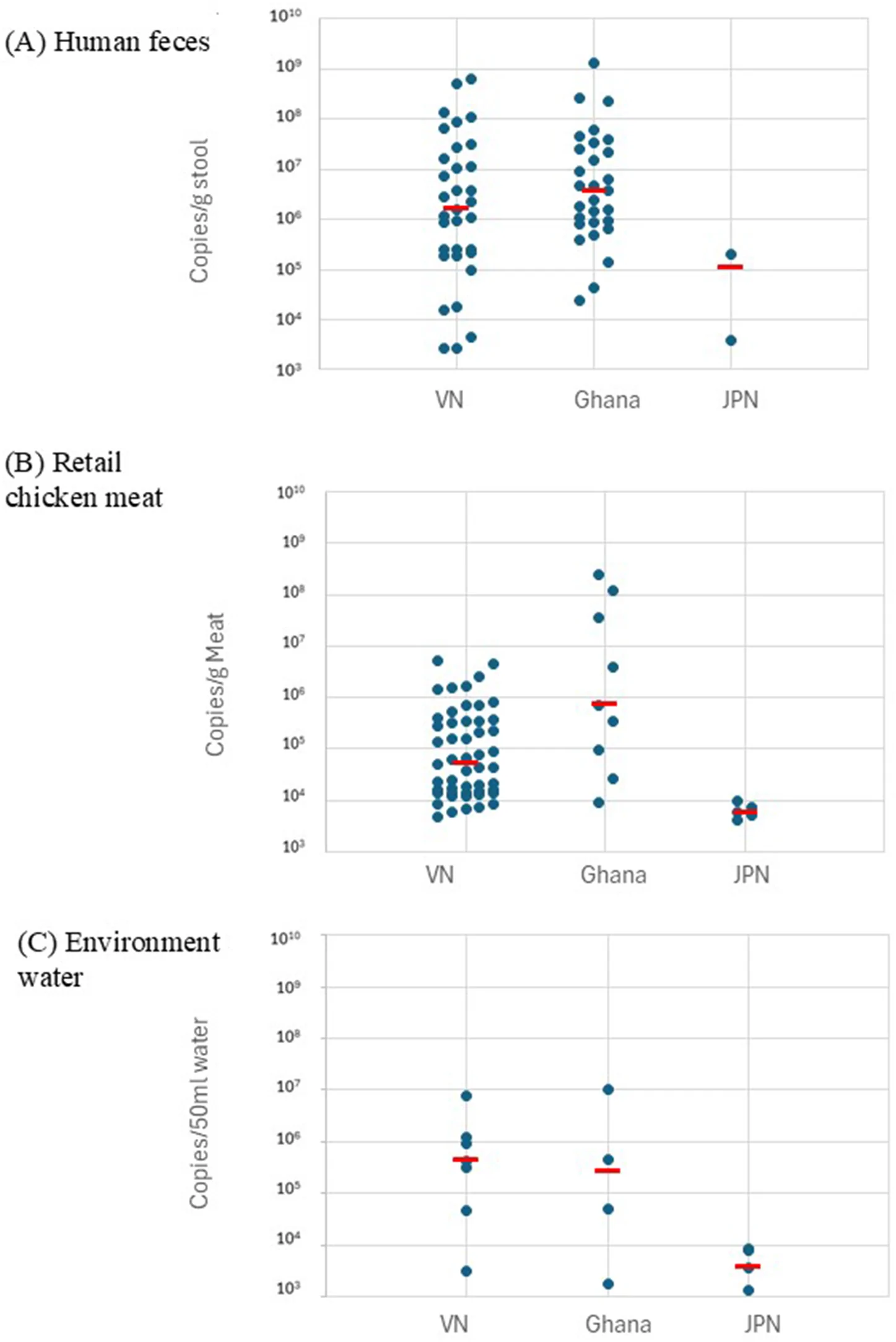
Levels of *merA* in human feces (A), chicken meat (B) and environment water (C). The dots in the figure indicate the *merA* level of each sample. Samples with copy numbers below 10³ are not shown. Red horizontal bars indicate the median.

The quantitative results of *merA* gene copies detected in various sample types are shown in Fig 1. In human fecal samples from Vietnamese residents, the number of *merA* gene copies per gram of feces varied widely between individuals, ranging from 10³ to as high as 10^8^ copies (median copy numbers: 1.578 × 10^6^). Similar variations were observed among Ghanaian residents, with copy numbers ranging from 10^4^ to 10^9^ copies/g (median copy numbers: 3.656 × 10^6^). In contrast, among Japanese residents, only two individuals tested positive for *merA*, with copy numbers limited to 10³ and 10⁵ (median copy numbers: 1.02 × 10^5^), respectively.

Comparable trends were observed in the retail chicken meat samples. In Japan, *merA* was detected in only 5 of 27 chicken samples, with copy numbers close to the detection limit (~10³). In sharp contrast, chicken samples from Vietnam and Ghana exhibited significantly higher copy numbers (*p* < 0.001), ranging from 10⁴ up to 10⁸ in Ghana.

A similar pattern was observed in environmental water samples. Although the detection rate of *merA* was comparably high across all three countries, the copy numbers showed notable differences: Japanese samples contained *merA* at levels of approximately 10³, whereas samples from Vietnam and Ghana reached up to 10⁷ copies, indicating substantially higher environmental burdens in those regions.

### Mercury concentrations in environmental water samples

The total Hg concentrations in eleven environmental water samples collected in Vietnam and Japan, as shown in Table 3, were measured using CV-AAS, and all samples contained <0.0005 mg/L of Hg.

### Isolation and characterization of mercury-resistant bacteria

**Fig 2:**
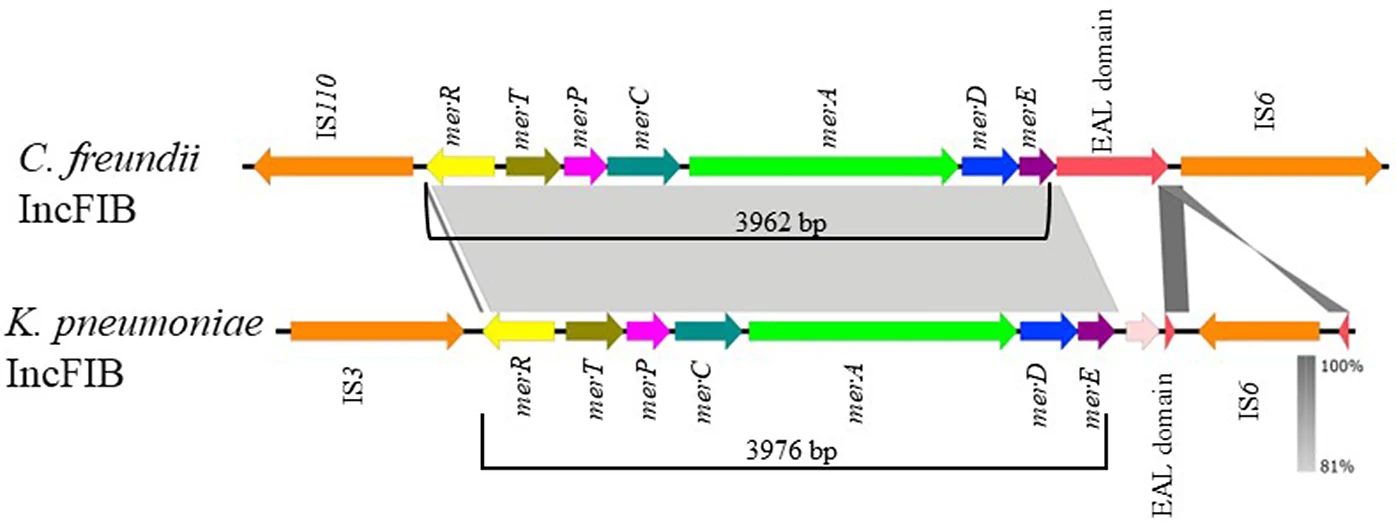
Comparison of the *mer* operon structure on the IncFIB plasmid of *C. freundii* TB25-009W-60-9 with that on the reference strain *Klebsiella pneumoniae* 1_GR_13. The numbers in the figure indicate the size of *mer* operon.

From environmental water samples in Vietnam and Ghana exhibiting high *merA* abundance, Hg-resistant bacteria were selectively isolated and characterized. The aim was to obtain representative isolates for species identification and genomic analysis, rather than to culture all *merA*-positive samples. Numerous colonies of Hg-resistant bacteria were growing even in the presence of a high Hg concentration (60 μM). Species identification of these colonies revealed a diverse range of bacterial species (Table 5). A representative strain, *C. freundii* TB25-009W-60–9, was chosen for genomic analysis to investigate the localization and structure of the *merA* gene. As a result, *merA* was localized on an IncFIB plasmid, and the structure of the *mer* operon, composed of other *mer* genes, was nearly identical to that of the reference strain *Klebsiella pneumoniae* 1_GR_13 [26] (Fig 2). This *mer* operon was flanked by two insertion sequences (ISs). No homology was found with most other genes, including the antimicrobial resistance genes, on the plasmid of the reference strain (S2Fig).

**Table 5:** Mercury-resistant strains isolated from environmental water in Vietnam and Ghana.

Country	Water sample ID	Strain ID	Organism
Vietnam	TB25-001W	TB25-001W-60-1	*Pseudomonas putida*
		TB25-001W-60-2	*Kluyvera cryocrescens*
		TB25-001W-60-4	*Klebsiella variicola*
	TB25-002W	TB25-002W-60-1	*Kluyvera cryocrescens*
		TB25-002W-60-4	*Raoultella ornithinolytica*
		TB25-002W-60-8	*Raoultella terrigena*
	TB25-003W	TB25-003W-60-2	*Klebsiella oxytoca*
		TB25-003W-60-3	*Leclercia adecarboxylata*
		TB25-003W-60-4	*Kluyvera cryocrescens*
		TB25-003W-60-5	*Aeromonas caviae*
	TB25-009W	TB25-009W-60-1	*Klebsiella oxytoca*
		TB25-009W-60-6	*Raoultella ornithinolytica*
		TB25-009W-60-8	*Enterobacter asburiae*
		TB25-009W-60-9	*Citrobacter freundii*
Ghana	Gha25-001W	Gha25-001W-2	*Escherichia coli*
		Gha25-001W-4	*Pseudomonas guariconensis*
		Gha25-001W-8	*Enterobacter hormaechei*
		Gha25-001W-9	*Citrobacter freundii*
		Gha25-001W-10	*Enterobacter cloacae*
		Gha25-001W-12	*Pseudomonas guariconensis*
	Gha25-003W	Gha25-003W-1	*Escherichia hermannii*
	Gha25-004W	Gha25-004W-1	*Acinetobacter towneri*
		Gha25-004W-2	*Enterobacter hormaechei*
		Gha25-004W-3	*Escherichia coli*
		Gha25-004W-4	*Klebsiella pneumoniae*
	Gha25-006W	Gha25-006W-1	*Priestia flexa*
		Gha25-006W-3	*Escherichia coli*

### Nucleotide sequence accession numbers

The complete genome sequence of *C. freundii* TB25-009W-60-9 has been deposited in DDBJ/ENA/GenBank under accession numbers AP040138 (chromosome) and AP040139 (plasmid).

## Discussion

The primer–probe set targeting *merA* demonstrated high sequence homology with a wide spectrum of *merA* gene sequences from diverse bacterial taxa, as revealed using BLAST analysis against GenBank. However, at least seven *merA* determinants have been documented [27], and the application of multiple primer–probe sets will be essential in future studies to ensure more comprehensive detection. Although the current approach is not exhaustive, it provides meaningful insights into the geographical distribution and prevalence of *merA*-harboring bacteria.

The detection of *merA* in fecal samples from residents and in retail domestic chicken meat suggests the presence of *merA*-harboring bacteria in the human gut microbiota and the food supply, respectively. These findings support the hypothesis that Hg-resistant bacteria may be transmitted through the food chain and may circulate through both environmental and dietary pathways. In this context, the presence of *merA* may serve as a potential microbiological indicator associated with Hg exposure via ingestion or environmental contact. However, *merA* should be interpreted not as a direct quantitative biomarker of individual Hg burden, but rather as an ecological marker reflecting environmental Hg exposure and the dissemination of Hg-resistant bacteria.

The prevalence of *merA* was higher in Ghana and Vietnam, but significantly lower in Japan. This disparity likely reflects differences in environmental Hg contamination, influenced by local industrial activities, mining practices, and regulatory frameworks. Although *merA* levels varied between individuals, overall fecal *merA* gene abundance was comparable between Vietnamese and Ghanaian residents. In contrast, only two low-level positives were detected among Japanese residents. A similar pattern was observed in chicken meat samples, suggesting shared exposure pathways between environmental and dietary sources.

While *merA* prevalence in environmental water was consistently high across all countries (75–100%), detection rates in human stools and chicken meat varied. For example, although 75% of Japanese water samples were *merA*-positive, the prevalence in human (6.8%) and chicken (18.5%) samples remained low. Conversely, in Ghana and Vietnam, high water prevalence corresponded with elevated detection rates in both human and chicken samples. These discrepancies suggest that, although aquatic environments may serve as reservoirs of *merA*-positive bacteria, their transmission into the food chain and human gut microbiota is modulated by additional factors. These may include dietary exposure pathways, environmental contamination levels, farming practices, food processing, and host-specific microbiome dynamics.

Thus, high *merA* prevalence in water may indicate a reservoir of potential risk, but its downstream impact on humans and food products likely depends on a complex interplay of ecological, cultural, and behavioral factors beyond environmental contamination alone.

Although Hg is a naturally occurring element and the *merA* gene is widely distributed, its relative abundance may act as an indicator of bioavailable Hg in the environment. Accordingly, the elevated levels of *merA* observed in Vietnam and Ghana are likely attributable to anthropogenic Hg contamination. In Ghana, artisanal and small-scale gold mining have been linked to Hg pollution [28], whereas in Vietnam, coal combustion and industrial discharge are probable contributors [29]. The lack of stringent regulatory controls and inadequate waste management in these countries may have exacerbated this issue. It is important to note that the WHO and national regulation in both Vietnam and Ghana specify a maximum permissible Hg concentration of 0.001 mg/L in drinking and surface water. In this study, all Vietnamese water samples contained total Hg levels below this regulatory threshold (<0.0005 mg/L). Nevertheless, the frequent detection of *merA* in these samples underscore the potential value of microbial indicators as complementary tools, since *merA* prevalence may reflect both current low-level exposure and historical Hg contamination even when physicochemical measurements fall below detection limits.

*merA* was detected in several samples where Hg was undetectable, suggesting that *merA* gene detection offers superior sensitivity for assessing environmental Hg exposure. Prior research supports this notion, highlighting that *merA*-positive bacteria may persist in environments with trace or undetectable levels of Hg, reflecting historical contamination or low-level chronic exposure [30].

Quantification was based on provisional copy number estimates using *E. coli* genomic DNA as a reference. Because *merA* is distributed across diverse bacterial taxa [31], with variable genomic localization (e.g., plasmid-borne or chromosomal) and copy numbers, the estimated values should be regarded as relative indices rather than absolute gene copy numbers. Furthermore, although an equal amount of each fecal sample was used for DNA extraction, no sample-specific normalization (e.g., to the 16S rRNA gene) was performed. Therefore, the present data are suitable for relative comparisons among samples and populations rather than for determining absolute *merA* abundance. Nevertheless, this approach provides a practical and consistent basis for cross-sample and cross-country comparisons.

The successful isolation and cultivation of *merA*-positive bacteria from water samples supports the validity of the molecular detection methods. Furthermore, the diversity of isolated Hg-resistant strains highlights the broad taxonomic range of *merA*-bearing organisms in aquatic systems.

In this study, *C. freundii* was isolated as a representative Hg-resistant bacterium, and its genome was analyzed to investigate the localization and structural characteristics of the *mer* operon, including *merA*. A comparative analysis revealed that the *mer* operon observed on the IncFIB plasmid of the isolated strain was nearly identical to that of the previously characterized *K. pneumoniae* 1_GR_13 [26]. The presence of insertion sequences flanking the *mer* operon further supports the potential for HGT. These findings are consistent with previous reports that *mer* operon is frequently associated with mobile genetic elements, facilitating their dissemination across diverse bacterial taxa and environmental niches [27]. The detection of nearly identical *mer* operon structures on plasmid IncFIB plasmids from different bacterial species suggested that plasmid-mediated HGT may play a significant role in the spread of Hg resistance determinants in aquatic environments.

The results of this study demonstrated that *merA* is a valuable biomarker for assessing Hg contamination. Furthermore, the application of this method to compare regional Hg pollution in Ghana and Vietnam with that in Japan highlights the potential of evaluating human fecal samples as a proxy for Hg exposure. From a public health standpoint, the hypothesis that human exposure to Hg or ingestion of Hg-resistant bacteria via food may lead to an increased prevalence of Hg-resistant bacteria within the gut microbiota represents a potentially sensitive and informative biomarker for assessing environmental health risks. This approach is supported by studies in wildlife, which have demonstrated correlations between fecal Hg levels and the composition of gut microbiota [32]. Future challenges include conducting qualitative assessments of the sources and historical progression of environmental Hg contamination in areas. Such assessments may be achieved through detailed analysis of the bacterial species composition of Hg-resistant strains and structural characterization of the *mer* operon, including the presence and types of associated ISs.

### Limitations

This study has several limitations. First, the limited sample size and restricted sampling area may limit the generalizability of the findings. Second, the primers and probe used may not detect all *merA* subtypes, potentially underestimating the overall prevalence of mercury-resistant bacteria. In addition, *merA* prevalence reflects bacterial adaptation to Hg selection pressure rather than Hg concentration itself. Hg-resistant bacteria may persist after Hg levels have declined following historical contamination or chronic low-level exposure; therefore, *merA* should be interpreted as an ecological indicator of Hg selection pressure rather than a direct proxy for the current Hg burden. The absence of direct Hg measurements in stool and meat samples also prevented quantitative assessment of the relationship between *merA* abundance and Hg concentrations. Future studies should include larger and more diverse sample sets, broader detection of *merA* subtypes, and paired analyses of Hg concentrations and *merA* prevalence to clarify the relationship among environmental exposure, bacterial resistance, and potential human health risks.

## Supporting information

S1 Table Primers and a probe for *merA.* (XLSX)

S2 Table Real-time PCR assay. (XLSX)

S3 Table *merA*-carrying bacterial species that showed 100% homology with the primers and probes used in this study. (XLSX)

S1 Fig (A) Standard curve of *merA* detected using real-time PCR. Cq, cycle threshold. (B) Real-time PCR amplification fluorescence curves of standard positive controls.
(PDF)

S2 Fig Comparative analysis of IncFIB plasmids between *Citrobacter freundii* TB25-009W-60–9 and reference *Klebsiella pneumoniae* 1_GR_13. Plasmid size: *C. freundii*200,468 bp; *K. pneumoniae* (reference)168,873 bp.
(PDF)

## References

[1] Lin Y, Wang S, Wu Q, Larssen T. Material Flow for the Intentional Use of Mercury in China. Environ Sci Technol. 2016;50(5):2337–44. doi: 10.1021/acs.est.5b04998 26812394

[2] Donkor AK, Bonzongo JC, Nartey VK, Adotey DK. Mercury in different environmental compartments of the Pra River Basin, Ghana. Sci Total Environ. 2006;368(1):164–76. doi: 10.1016/j.scitotenv.2005.09.046 16243381

[3] Clarkson TW, Magos L. The toxicology of mercury and its chemical compounds. Crit Rev Toxicol. 2006;36(8):609–62. doi: 10.1080/10408440600845619 16973445

[4] Scheuhammer AM, Meyer MW, Sandheinrich MB, Murray MW. Effects of environmental methylmercury on the health of wild birds, mammals, and fish. Ambio. 2007;36(1):12–8. doi: 10.1579/0044-7447(2007)36[12:eoemot]2.0.co;2 17408187

[5] Priyadarshanee M, Chatterjee S, Rath S, Dash HR, Das S. Cellular and genetic mechanism of bacterial mercury resistance and their role in biogeochemistry and bioremediation. J Hazard Mater. 2022;423(Pt A):126985. doi: 10.1016/j.jhazmat.2021.126985 34464861

[6] World Health Organization. Guidelines for drinking-water quality: fourth edition incorporating the first addendum. Geneva: World Health Organization. 2017. 28759192

[7] Ministry of Natural Resources and Environment V. National technical regulation on hazardous waste management – QCVN 08:2023/BTNMT. Hanoi: Government of Vietnam. 2023. https://datafiles.chinhphu.vn/cpp/files/vbpq/2023/3/01-btnmt-qc08.pdf

[8] Ghana Standards Authority. Drinking water specification – Part 1: Physical and chemical requirements (GS 175-1:2021). Accra: Ghana Standards Authority. 2021. https://www.gsa.gov.gh/wp-content/uploads/2021/03/DGS-175_2021.pdf

[9] Peng X, Yang Y, Yang S, Li L, Song L. Recent advance of microbial mercury methylation in the environment. Appl Microbiol Biotechnol. 2024;108(1):235. doi: 10.1007/s00253-023-12967-6 38407657 PMC10896945

[10] Osborn AM, Bruce KD, Strike P, Ritchie DA. Distribution, diversity and evolution of the bacterial mercury resistance (mer) operon. FEMS Microbiol Rev. 1997;19(4):239–62. doi: 10.1111/j.1574-6976.1997.tb00300.x 9167257

[11] Barkay T. Adaptation of aquatic microbial communities to hg stress. Appl Environ Microbiol. 1987;53(12):2725–32. doi: 10.1128/aem.53.12.2725-2732.1987 16347488 PMC204188

[12] Boyd ES, Barkay T. The mercury resistance operon: from an origin in a geothermal environment to an efficient detoxification machine. Front Microbiol. 2012;3:349. doi: 10.3389/fmicb.2012.00349 23087676 PMC3466566

[13] Agarwal M, Rathore RS, Jagoe C, Chauhan A. Multiple Lines of Evidences Reveal Mechanisms Underpinning Mercury Resistance and Volatilization by Stenotrophomonas sp. MA5 Isolated from the Savannah River Site (SRS), USA. Cells. 2019;8(4):309. doi: 10.3390/cells8040309 30987227 PMC6523443

[14] Frossard A, Donhauser J, Mestrot A, Gygax S, Bååth E, Frey B. Long- and short-term effects of mercury pollution on the soil microbiome. Soil Biology and Biochemistry. 2018;120:191–9. doi: 10.1016/j.soilbio.2018.01.028

[15] Nakamura K, Sakata T, Nakahara H. Volatilization of mercury compounds by methylmercury-volatilizing bacteria in Minamata Bay sediment. Bull Environ Contam Toxicol. 1988;41(5):651–6. doi: 10.1007/BF02021014 3233363

[16] Nazaret S, Jeffrey HW, Saouter E, Von Haven R, Barkay T. merA gene expression in aquatic environments measured by mRNA production and Hg (II) volatilization. Applied and Environmental Microbiology. 1994;60(11):4059–65. doi: 10.1128/aem.60.11.4059-4065 7527625 PMC201936

[17] Yamamoto Y, Kawahara R, Fujiya Y, Sasaki T, Hirai I, Khong DT, et al. Wide dissemination of colistin-resistant Escherichia coli with the mobile resistance gene mcr in healthy residents in Vietnam. J Antimicrob Chemother. 2019;74(2):523–4. doi: 10.1093/jac/dky435 30380052

[18] Le YH, Hoang HTT, Khong DT, Nguyen TN, Que TA, Pham DT, et al. Contamination of retail market meat with extended-spectrum beta-lactamase genes in Vietnam. Int J Food Microbiol. 2025;430:111061. doi: 10.1016/j.ijfoodmicro.2025.111061 39827751

[19] Yamamoto Y, Kawahara R, Fujiya Y, Sasaki T, Hirai I, Khong DT, et al. Wide dissemination of colistin-resistant Escherichia coli with the mobile resistance gene mcr in healthy residents in Vietnam. J Antimicrob Chemother. 2019;74(2):523–4. doi: 10.1093/jac/dky435 30380052

[20] Ministry of the Environment, Japan. Method for evaluating water quality monitoring results in public water areas (in Japanese). Tokyo: Ministry of the Environment. 2023. https://www.env.go.jp/content/900405217.pdf

[21] SciencePrimer. Copy number calculator for real-time PCR. https://scienceprimer.com/copy-number-calculator-for-realtime-pcr Accessed 2026 January 15.

[22] Matsui K, Yoshinami S, Narita M, Chien M-F, Phung LT, Silver S, et al. Mercury resistance transposons in Bacilli strains from different geographical regions. FEMS Microbiol Lett. 2016;363(5):fnw013. doi: 10.1093/femsle/fnw013 26802071

[23] DDBJ Center. DFAST: DDBJ Fast Annotation and Submission Tool. National Institute of Genetics; [cited 2026 Jan 15]. Available from: https://dfast.ddbj.nig.ac.jp/dfc/

[24] Center for Genomic Epidemiology. ResFinder 4.1: Identification of acquired antimicrobial resistance genes. https://cge.food.dtu.dk/services/ResFinder/ Accessed 2026 January 15.

[25] Center for Genomic Epidemiology. PlasmidFinder 2.1: Identification of plasmid replicons in bacterial genomes. https://cge.food.dtu.dk/services/PlasmidFinder/ Accessed 2026 January 15.

[26] Pitt ME, Elliott AG, Cao MD, Ganesamoorthy D, Karaiskos I, Giamarellou H, et al. Multifactorial chromosomal variants regulate polymyxin resistance in extensively drug-resistant Klebsiella pneumoniae. Microb Genom. 2018;4(3):e000158. doi: 10.1099/mgen.0.000158 29431605 PMC5885010

[27] Møller AK, Barkay T, Hansen MA, Norman A, Hansen LH, Sørensen SJ, et al. Mercuric reductase genes (merA) and mercury resistance plasmids in High Arctic snow, freshwater and sea-ice brine. FEMS Microbiol Ecol. 2014;87(1):52–63. doi: 10.1111/1574-6941.12189 23909591

[28] Addai-Arhin S, Novirsa R, Jeong H, Phan QD, Hirota N, Ishibashi Y, et al. Mercury waste from artisanal and small-scale gold mining facilities: a risk to farm ecosystems-a case study of Obuasi, Ghana. Environ Sci Pollut Res Int. 2023;30(2):4293–308. doi: 10.1007/s11356-022-22456-4 35969344

[29] Dat ND, Truong MT, Nguyen LSP, Kim Tran AT, Duc NM, Vo T-D-H, et al. Street dust mercury levels among different land-use categories in Ho Chi Minh city, Vietnam: Source apportionment and risk estimation. Atmospheric Pollution Research. 2023;14(1):101623. doi: 10.1016/j.apr.2022.101623

[30] Narita M, Matsui K, Huang C-C, Kawabata Z, Endo G. Dissemination of TnMERI1-like mercury resistance transposons among Bacillus isolated from worldwide environmental samples. FEMS Microbiol Ecol. 2004;48(1):47–55. doi: 10.1016/j.femsec.2003.12.011 19712430

[31] Christakis CA, Barkay T, Boyd ES. Expanded Diversity and Phylogeny of mer Genes Broadens Mercury Resistance Paradigms and Reveals an Origin for MerA Among Thermophilic Archaea. Front Microbiol. 2021;12:682605. doi: 10.3389/fmicb.2021.682605 34248899 PMC8261052

[32] Rothenberg SE, Sweitzer DN, Rackerby BR, Couch CE, Cohen LA, Broughton HM, et al. Fecal Methylmercury Correlates With Gut Microbiota Taxa in Pacific Walruses (Odobenus rosmarus divergens). Front Microbiol. 2021;12:648685. doi: 10.3389/fmicb.2021.648685 34177830 PMC8220164

