# Supplementary material for: Comparative prevalence of the mercury resistance gene merA in human feces, food, and environmental water from Japan, Vietnam, and Ghana
Source: PLoS One. 2026 Sep 8;21(9):e0357976. doi: 10.1371/journal.pone.0357976 (PMC13552768; doi:10.1371/journal.pone.0357976)
Supplement: Supplementary file 1 [file pone.0357976.s004.pdf]

(A)

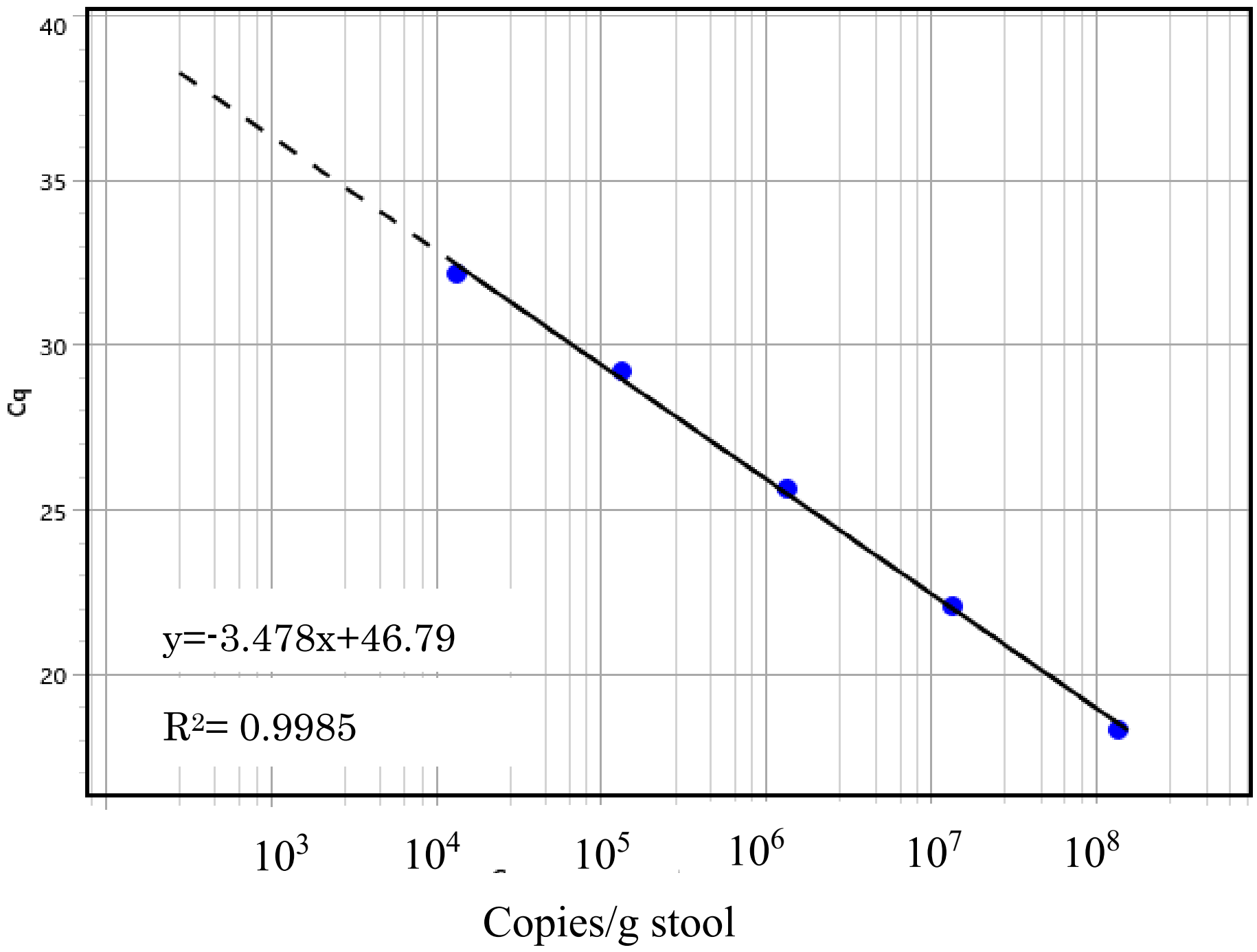

(B)

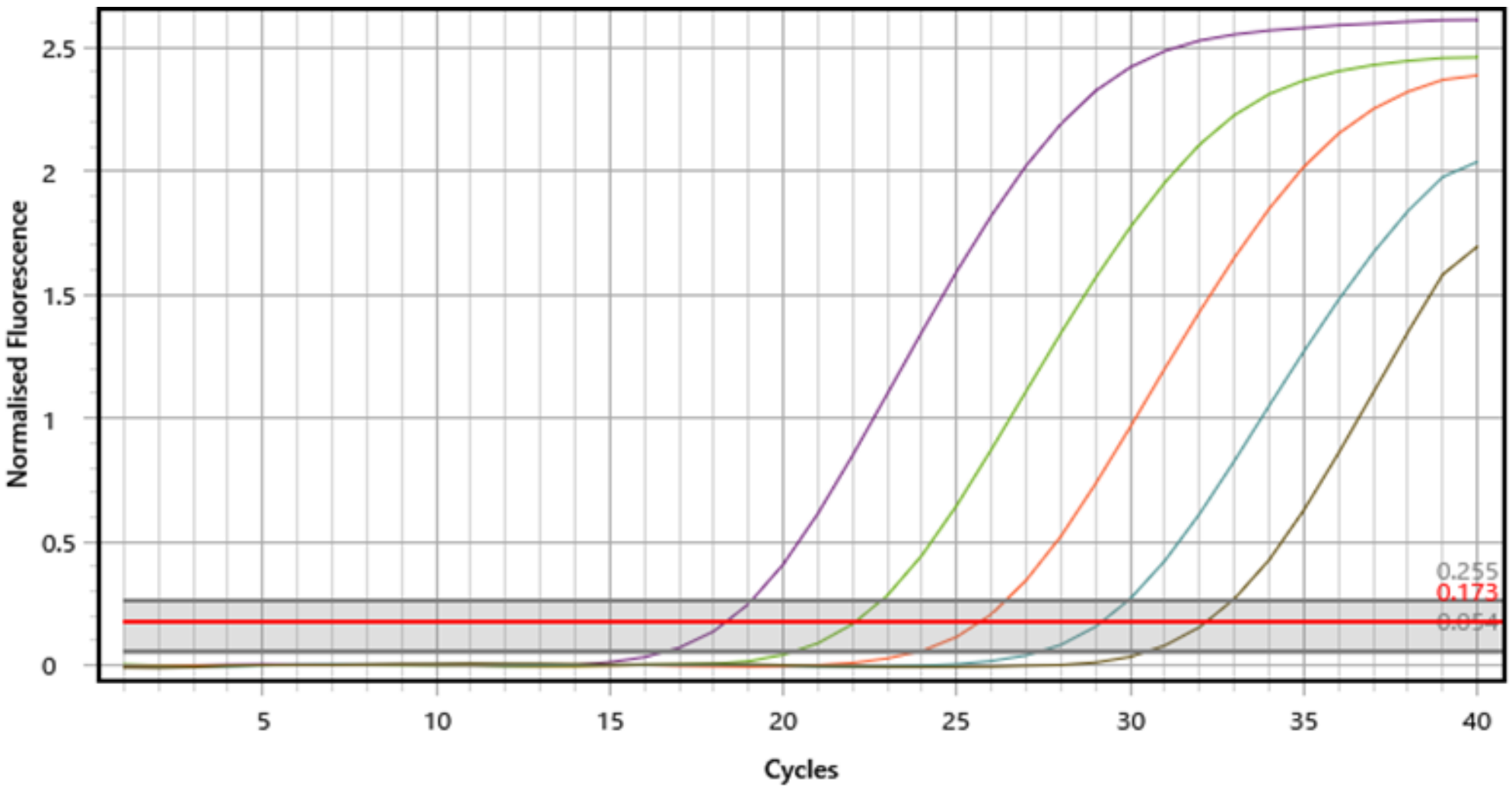

Fig S1. (A) Standard curve of *merA* detected using real-time PCR. Cq, cycle threshold. (B) Real-time PCR amplification fluorescence curves of standard positive controls.
