# Supplementary material for: Comparative prevalence of the mercury resistance gene merA in human feces, food, and environmental water from Japan, Vietnam, and Ghana
Source: PLoS One. 2026 Sep 8;21(9):e0357976. doi: 10.1371/journal.pone.0357976 (PMC13552768; doi:10.1371/journal.pone.0357976)
Supplement: Supplementary file 2 [file pone.0357976.s005.pdf]

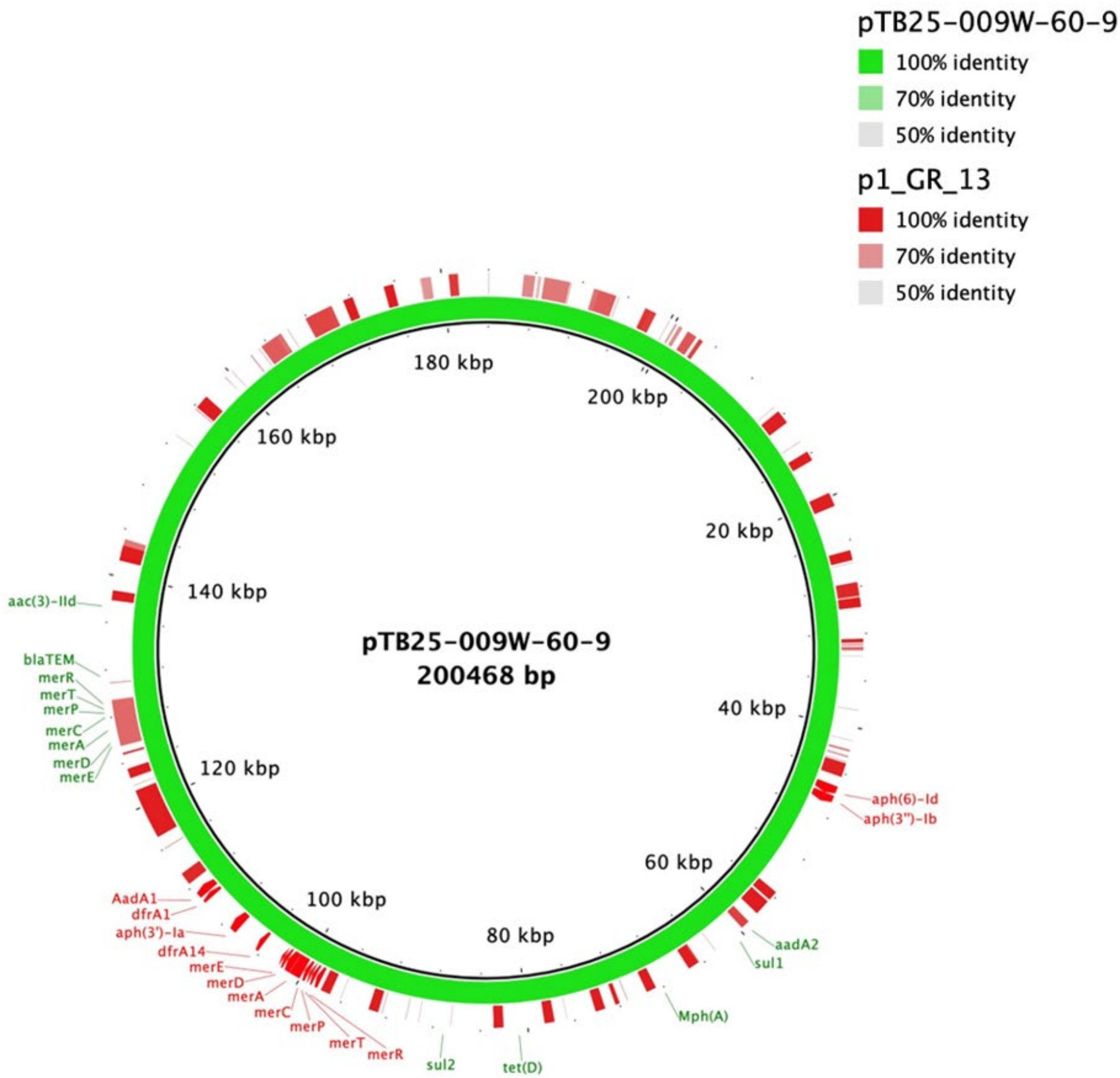

Fig S2. Comparative analysis of IncFIB plasmids between *Citrobacter freundii* TB25-009W-60-9 and reference *Klebsiella pneumoniae* 1_GR_13. Plasmid size: *C. freundii* – 200,468 bp; *K. pneumoniae* (reference) – 168,873 bp.
