# Supplementary material for: Comparative prevalence of the mercury resistance gene merA in human feces, food, and environmental water from Japan, Vietnam, and Ghana
Source: PLoS One. 2026 Sep 8;21(9):e0357976. doi: 10.1371/journal.pone.0357976 (PMC13552768; doi:10.1371/journal.pone.0357976)
Supplement: Supplementary file 3 [file pone.0357976.s007.docx]

Comments to the Author:

The authors investigated the prevalence and abundance of the mercury resistance gene merA in human feces, retail chicken meat, and environmental water samples collected from Japan, Vietnam, and Ghana. Although the current approach is not exhaustive, it provides meaningful insights into the geographical distribution and prevalence of merA-harboring bacteria. The revised version manuscript was evaluated thoroughly, and while the draft has been significantly improved, there are minor concerns related to formatting. Please find my comments below:

Comments:

1. Line 66, 67, 68: Please cite in proper referencing format rather than links.
2. Please confirm referencing style in both figure captions as well as in body.
3. Line 149, 174, 204,206,207: Please use proper citation style.
4. Line 227: It should be Fig. S1 and not S1 Fig (Please check journal format if needed). Same comment for Line 222, 227,288.
