# Supplementary material for: Comparative prevalence of the mercury resistance gene merA in human feces, food, and environmental water from Japan, Vietnam, and Ghana
Source: PLoS One. 2026 Sep 8;21(9):e0357976. doi: 10.1371/journal.pone.0357976 (PMC13552768; doi:10.1371/journal.pone.0357976)
Supplement: Supplementary file 4 [file pone.0357976.s008.docx]

**PONE-D-25-30012**

Multinational comparison of the prevalence of the mercury resistance biomarker gene *merA* in human feces, food, and environmental water

**PLOS ONE**

**Response to the comments**

**Journal requirements:**

**1. Please ensure that your manuscript meets PLOS ONE's style requirements, including those for file naming.**

►Response:

Yes, we have checked that the file names you mentioned match the required style.

**2. Please include a complete copy of PLOS’ questionnaire on inclusivity in global research in your revised manuscript**.

►Response:

We have attached the questionnaire as per your suggestion.

**3. In your Methods section, please provide additional information regarding the permits you obtained for the work.**

►Response:

We added it as per your suggestion (Line 95-99, 102-106).

**4. 5. Please state what role the funders took in the study.**

►Response:

We removed the funding-related text from the paper and stated the role the funders took in the study in Financial Disclosure.

**6. Please include a separate caption for each figure in your manuscript.**

►Response:

Yes, we did as per your suggestion (Line 255-257, 290-292).

**7. Please remove all personal information, ensure that the data shared are in accordance with participant consent.**

►Response:

Yes, we have checked to make sure it does not contain any personal information.

**8. If the reviewer comments include a recommendation to cite specific previously published works, please review and evaluate these publications to determine whether they are relevant and should be cited.**

►Response:

Yes, we have confirmed this.

**9. Please review your reference list to ensure that it is complete and correct.**

►Response:

Yes, we have checked that the references are properly cited.

**Reviewer #1**

**The authors attempt to the prevalence and abundance of the mercury resistance gene *merA* in human feces, retail chicken meat, and environmental water samples collected from Japan, Vietnam, and Ghana. The work is important because the main focus is on the mercury resistance gene *merA* as a biomarker for regional environmental mercury contamination. ----------------.**

►**Response:**

We sincerely thank the reviewer for your positive and encouraging comments regarding our manuscript.

**Reviewer #2**:

**Overall, the paper is well written, but I suggest the author emphasize the public health implications of mercury contamination more strongly in the introduction and discussion sections.**

►**Response:**

We sincerely thank the reviewer for the constructive and thoughtful feedback provided. In accordance with the reviewer’s valuable feedback, we have included additional information in the Introduction on the potential health impacts of mercury contamination (Line 56-57).

**Comment 1**:

**As authors acknowledge that mercury is a naturally occurring substance, it would be beneficial to include information about the acceptable threshold levels that are not hazardous to human health, as well as the points at which these levels become harmful. Additionally, it’s worth noting that the levels of mercury observed in Ghana and Vietnam exceed these thresholds.**

►**Response:**

Thank you for this important suggestion. We appreciate your insightful comment regarding the need to include information on acceptable mercury threshold levels. As suggested, we have added information on acceptable threshold levels of mercury in water and compared them with the levels reported in Ghana and Vietnam. According to the WHO guidelines, the permissible limit of mercury in drinking water is 0.001 mg/L. The national regulation of both Vietnam and Ghana adopt the same threshold of 0.001 mg/L for drinking and surface water. We have now incorporated this information into the Introduction and Discussion to emphasize the public health significance of our findings (Line 64-71, 342-349).

**Comment 2**:

**There seems to be a slight variation in the prevalence of mercury in water compared to human and chicken sample across different countries. I would appreciate clarification on this discrepancy in discussion.**

►**Response**:

We thank the reviewer for this insightful comment. Indeed, the prevalence of *merA* in water did not always align with human stool or chicken samples across countries. We have now expanded our discussion to clarify this point in the revised manuscript as suggested by the reviewer (Line 322-331).

**Comment 3**:

**Methodology section lacks clarify regarding which experiments were conducted for various samples. It appears that culture techniques were only applied to water samples, but need a justification why it was not applied to other samples? A flow diagram would enhance understanding of the methodology used.**

►**Response**:

We sincerely thank the reviewer for this insightful comment. In accordance with the reviewers’ comments, we have clarified in the revised manuscript the rationale for using only environmental water in the culture experiments. We hope that this addition appropriately addresses the reviewer’s comment (Line 276-279).

The sample processing procedures used in this study are relatively simple, and we believe that the experiments can be readily reproduced based on the information provided in the Methods section. If the inclusion of a scheme diagram is deemed necessary, we would be happy to provide one upon your further instruction.

**Comment 4:**

**Statistical analysis is not explicitly explained whether it was bivariate or multivariate analysis. If the number of samples is less than 30, which statistical test was performed.**

►**Response**:

Thank you for pointing this out. Statistical analyses in our study were conducted as bivariate comparisons. We have clarified this point in the revised Statistical analysis subsection of the Materials and methods (Line 214-215).

**Comment 5**:

**In Table 5, some samples were not tested any methods. Could the authors explain the reason for this?**

►**Response**:

We appreciate the reviewer’s comment. All environmental water samples were first tested for the presence of the *merA* gene by real-time PCR. For subsequent culture work, we focused on a subset of *merA*-positive samples in order to obtain representative mercury-resistant bacterial isolates for species identification and genomic characterization. This approach was taken because the objective was not to culture every *merA*-positive sample, but rather to demonstrate the diversity of mercury-resistant bacteria present and to obtain representative strains for further genomic analysis (Line 276-279). Table 5 therefore shows only those isolates that were successfully cultured and identified.

**Comment 6**:

**I recommend rewriting lines 189 to 194 to clarify. It would be helpful to specify which databases or software were used for annotation, the purpose of utilizing ResFinder (e.g., for investigating the *merA* gene in silico), and the reason for using Geneious Prime.**

►**Response**:

We thank the reviewer for this valuable suggestion. We have revised the text to more clearly describe the genome annotation and analysis process. Specifically, we now state that genome annotation was performed using the DFAST tool, resistance genes were investigating with ResFinder, plasmid replicons were identified using PlasmidFinder, and Geneious Prime was employed for sequence visualization and comparative analysis of gene structure (Line 204-210).

**Comment 7**:

**In line 55, please cite the reference using a numerical format rather than including a hyperlink.**

►**Response**:

We thank the reviewer for pointing out the inconsistency. In line 55, we had included both numerical reference [2] and hyperlink, which referred to a different source. Since our intended citation is Reference [2] (Donkor et al., 2006), we have removed the hyperlink to ensure consistency with the numerical citation format required by the journal (Line 55).

**Comment 8**:

**There should be a consistent structure applied to all tables. Overall, the formatting quality of the table is poor.**

►**Response**:

We appreciate the reviewer’s suggestion. We have taken care to confirm that all tables in the manuscript adhere to the submission requirements and have made appropriate adjustments where necessary.

**Comment 9**:

**Figure 1 does not clearly indicate which dots correspond to which countries. Improvements in clarity would be beneficial.**

►**Response**:

The incorrect positioning of the country names in Figure 1 seems to have been caused by the PDF conversion process. In the revised version, we will double-check to ensure all labels are displayed correctly. Thank you very much for your helpful comment.

Once again, we appreciate reviewer’s valuable comments, which have helped us improve both the clarity and scientific rigor of the manuscript.
