# Supplementary material for: Comparative prevalence of the mercury resistance gene merA in human feces, food, and environmental water from Japan, Vietnam, and Ghana
Source: PLoS One. 2026 Sep 8;21(9):e0357976. doi: 10.1371/journal.pone.0357976 (PMC13552768; doi:10.1371/journal.pone.0357976)
Supplement: Supplementary file 5 [file pone.0357976.s009.docx]

**PONE-D-25-30012**

“Multinational comparison of the prevalence of the mercury resistance biomarker gene *merA* in human feces, food, and environmental water”

**Response to the comments**

**1.	Line 66, 67, 68: Please cite in proper referencing format rather than links.**

►Response: As suggested, we have reformatted the cited web addresses according to the PLOS ONE reference style.

**2.	Please confirm referencing style in both figure captions as well as in body.**

►Response: In accordance with the reviewer’s suggestion, we have unified the citation style throughout the manuscript.

**3.	Line 149, 174, 204,206,207: Please use proper citation style.**

►Response: we have reformatted the cited web addresses according to the PLOS ONE reference style.

**4.	Line 227: It should be Fig. S1 and not S1 Fig (Please check journal format if needed). Same comment for Line 222, 227,288.**

►Response: In accordance with the reviewer’s comment, we revised “S1 Fig” to “Fig. S1” and made the same correction throughout the manuscript.
