# Supplementary material for: Comparative prevalence of the mercury resistance gene merA in human feces, food, and environmental water from Japan, Vietnam, and Ghana
Source: PLoS One. 2026 Sep 8;21(9):e0357976. doi: 10.1371/journal.pone.0357976 (PMC13552768; doi:10.1371/journal.pone.0357976)
Supplement: Supplementary file 6 [file pone.0357976.s010.docx]

**PONE-D-25-30012**

Manuscript title: “***Multinational comparison of the prevalence of the mercury resistance biomarker gene merA in human feces, food, and environmental water***”.

We sincerely thank the Editor and Reviewer for this careful evaluation of our manuscript and for the constructive and detailed comments, which have helped improve the clarity of our study. We have revised the manuscript extensively in response to all points raised.

Below we provide a point-by-point response.

**I. Response to Editor’s comment:**

**Comment 1**: *Can you provide statistical correlation analysis between merA copy numbers and measured mercury concentrations in water samples to support your biomarker claim?*

►**Response**:

We appreciate the suggestion to perform a statistical correlation analysis. However, we found that a formal correlation analysis between *merA* copy numbers and measured mercury concentrations was not feasible for this study.

Total mercury concentrations were measured in 11 environmental water samples from Vietnam and Japan, and all samples contained <0.0005 mg/L of Hg. Because these values showed minimal variability and clustered below the detection limit, falling well below the regulatory threshold of 0.001 mg/L, there was insufficient numerical variance to support a meaningful statistical correlation.

Despite these undetectable physicochemical levels, the *merA* gene was frequently detected in the same samples. This finding underscores the potential of *merA* as a more sensitive biological indicator, capable of reflecting low-level chronic exposure or historical contamination even when traditional mercury measurements fall below detection limits.

**Comment 2**: *Why was mercury speciation (e.g., methylmercury vs. inorganic mercury) not performed, given that MeHg is the most toxic and biologically relevant form?*

►**Response**:

We thank to Editor with the important comment regarding mercury speciation. While MeHg represents the most toxic and biologically relevant form of mercury for higher organisms, the primary objective of this study was to evaluate the distribution of the mercury resistance gene *merA* as a microbial biomarker of mercury contamination rather than to characterize mercury speciation in the environment.

The *merA* gene encodes mercuric reductase, which reduces inorganic mercury [Hg(II)] to elemental mercury (Hg⁰) and serves as a key marker of bacterial resistance to inorganic mercury. In contrast, MeHg degradation mainly involves *merB*, which was outside the scope of this study. Therefore, mercury speciation analysis was not included. Future studies incorporating mercury speciation and additional *mer*-related genes, including *merB*, would help clarify microbial responses to different mercury forms.

**Comment 3**: *Why were mercury concentration not measured in Ghanaian water samples, and how can geographic comparisons be made without these data?*

►**Response**:

We thank the Editor for raising this important point. Mercury concentrations were not measured in the Ghanaian water samples because they were collected during a later field survey focused primarily on microbial mercury resistance rather than environmental mercury quantification. In contrast, chemical mercury measurements were conducted for samples from Vietnam and Japan as part of the original study design. Because mercury concentrations were below the detection limit in both the Vietnamese samples with high *merA* levels and the Japanese samples with low *merA* levels, we believe that the lack of mercury measurement data for the Ghanaian samples is unlikely to materially affect the overall rationale of the study.

**Comment 4**: *If fecal merA is proposed as a proxy for human mercury expose, why were mercury levels not directly measured in human feces, blood, hair, or urine samples?*

►**Response:**

We appreciate the Editor’s important observation. The primary objective of this study was to investigate the prevalence and distribution of the mercury resistance gene *merA* within the human gut microbiota and environmental samples, and to explore its potential relevance as a microbiological indicator of mercury exposure. This work was designed as a microbial gene surveillance study rather than a human biomonitoring investigation. Accordingly, direct measurements of mercury concentrations in feces, blood, hair, or urine were not included in the present study design. We agree that correlation with established biomarkers of human mercury exposure would be necessary to validate fecal *merA* as a quantitative proxy of individual mercury burden within the gut environment.

To clarify the scope of the study, we have revised the Discussion to emphasize that fecal *merA* detection should be interpreted as a potential microbial indicator reflecting environmental or dietary mercury exposure at the community level, rather than as a validated clinical biomarker of individual mercury burden.

**Comment 5**: *Why were mercury concentrations not determined in chicken meat to validate the proposed food-chain transmission hypothesis?*

►**Response**:

We thank the Editor for this valuable comment. The objective of this study was to assess the prevalence of the mercury resistance gene *merA* within microbial communities present in environmental water, chicken meat, and human fecal samples. Accordingly, the analytical focus for chicken samples was on bacterial DNA extraction and molecular detection of resistance determinants rather than on quantification of mercury residues in the food matrix.

We agree that direct measurement of mercury concentrations in chicken meat would provide valuable complementary information and could further strengthen evaluation of potential food-chain transmission pathways. However, the present study was designed to investigate microbial resistance gene distribution as an ecological indicator associated with environmental mercury exposure, rather than to conduct a food toxicology assessment.

**Comment 6**: *How do you do you account for the relatively small and uneven sample sizes across the countries (e.g., Ghana chicken n=10)? Was a power calculation performed?*

►**Response**:

We thank the Editor for this important methodological consideration. We recognize that the sample sizes were relatively small and uneven across countries, particularly for Ghanaian chicken samples. This study was designed as a comparative molecular surveillance investigation to explore the distribution patterns of the *merA* gene across different ecological compartments and geographic regions. Therefore, formal power calculations were not performed prior to sampling.

We agree that the limited and uneven sample sizes reduce constraints of the generalizability of the finding. Accordingly, the geographic trends observed should be considered exploratory rather than definitive. We have xplicitly acknowledged this limitation and emphasized that larger, balanced studies will be required to confirm and quantify the observed regional differences.

**Comment 7**: *Why were merA copy numbers not normalized to total bacterial abundance (e.g., 16S rRNA gene copies) to control for differences in microbial biomass?*

►**Response**:

We thank the Editor for this observation. Normalization of *merA* copy numbers to total bacterial abundance would indeed allow calculation of relative abundance and better control for variation in microbial biomass across samples. In the present study, *merA* was quantified as absolute copy numbers per extraction volume to provide a comparative index of gene prevalence across environmental, food, and fecal matrices. The primary objective was to assess distribution patterns rather than to characterize microbiome composition or relative gene abundance within bacterial communities.

**Comment 8**: *How do you justify the use of E. coli genomic DNA as a universal standard for merA quantification, given variability in genome size and plsamid copy number among species?*

►**Response:**

We thank the Editor reviewer for this important point. In this study, *E. coli* genomic DNA containing *merA* was used as a calibration reference to generate a standard curve for qPCR quantification. *merA* is distributed across diverse bacterial species and may be located either chromosomally or on plasmids with variable copy numbers. Therefore, the resulting values should not be interpreted as absolute gene copy numbers across species.

In addition, *merA* quantities are reported as *E. coli* genome-equivalent copies, providing a standardized and internally consistent reference point for calculating relative copy numbers across different matrices. This approach allows relative comparison of *merA* abundance between matrices and geographic regions, while the true gene copy numbers may vary depending on host genome structure and plasmid content.

**Comment 9**: *Why were Cq values outside the standard curve range extrapolated, and how might this affect quantitative?*

►**Response:**

We thank the Editor for this critical technical observation. A small number of samples produced Cq values slightly outside the validated range of the standard curve, primarily at the low-copy-number end. These signals were near the limit of detection and represent low-abundance *merA* positivity. To assess potential bias, we evaluated the impact of including versus excluding these samples in downstream analyses. Exclusion of these low-positive, out-of-range samples did not alter the overall prevalence patterns, geographic trends, or statistical interpretations. Therefore, while quantitative precision at these extremes may be reduced, their inclusion does not materially affect the study conclusions.

**Comment 10**: *Given that at least seven merA exist, how do you address potential underestimation due to using only a single primer-probe set?*

►**Response:**

We thank the Editor for raising the valuable point. It is well established that multiple *merA* determinants exist, and the use of a single primer–probe set may not capture the full genetic diversity of mercury resistance genes. To mitigate this risk, we performed BLAST analysis against GenBank to confirm high sequence homology with a broad range of commonly reported *merA* sequences across diverse bacterial taxa. The selected primer–probe set demonstrated 100% homology with multiple representative species (high specificity toward sequences from over 12 bacterial species), supporting its broad applicability.

Nevertheless, we acknowledge that certain divergent or less-characterized *merA* variants may not be detected by this assay, potentially leading to underestimation of total *merA* prevalence.

We have explicitly acknowledged this limitation and emphasized identified the development of multiplex assays targeting multiple *merA* subtypes as an important direction for future research to achieve more comprehensive surveillance (Limitations).

**Comment 11**: *Does detection of merA DNA confirm functional resistance? How you performed RT-qPCR or phenotypic assays to confirm gene expression or enzymatic activity?*

►**Response:**

We thank the Editor for this important mechanistic clarification. Detection of merA DNA alone does not directly demonstrate active gene expression or enzymatic activity. The qPCR analysis performed in this study therefore indicates the **presence and distribution of the genetic determinant**, but does not by itself confirm functional mercury reduction within the microbial communities.

To partially address functional relevance, we performed phenotypic isolation of mercury-resistant bacteria using HgCl₂-supplemented media and subsequently confirmed the presence of *merA* in representative isolates. The recovery of viable isolates from merA-positive samples supports the biological relevance of the molecular detection results and indicates that the detected gene is associated with phenotypically resistant organisms.

However, **RT-qPCR or direct enzymatic assays measuring Hg(II) reductase activity were not performed** in this study, as the primary objective was to assess the **distribution and prevalence of** merA across different sample matrices rather than to characterize gene expression dynamics.

**Comment 12**: *Why were potential confounding variables in human participants (dietary habits, fish consumption, occupational expose, antibiotic use, age, sex) not analyzed?*

►**Response:**

We thank the Editor for this insightful observation. The individual level variables such as dietary habits, including fish consumption, occupational exposure, antibiotic use, age, and sex may influence gut microbiota composition and potentially affect *merA* prevalence in human fecal samples. However, the primary objective of this study was to conduct a comparative molecular survey of *merA* distribution across environmental, food, and human samples at the population level across different ecological matrices, rather than investigating individual level determinants of *merA* carriage.

A detailed epidemiological data were not incorporated into the analytical framework, and therefore multivariable assessment of potential confounding factors was not performed. Accordingly, fecal *merA* detection should be interpreted as reflecting broader environmental and community-level exposure contexts rather than as a direct measure of individual mercury burden or personalized risk.

**Comment 13**: *How do you explain the high prevalence of merA in Japanese environmental despite low detection in human feces and chicken meat?*

►**Response**:

We appreciate this insightful observation. The low *merA* levels observed in Japanese environmental water samples may partly explain the low prevalence of *merA* in human fecal and chicken meat samples; however, the relationship remains unclear. The prevalence of *merA* in the human gut microbiota is likely influenced by multiple factors in addition to environmental exposure. Therefore, the extent to which environmental water serves as a reservoir contributing to human or food-chain exposure remains to be determined and warrants further investigation.

**Comment 14**: *Can you clarify how a cross-sectional study design allows inference regarding transmission pathways (environment → food → human)?*

►**Response**:

We appreciate this insightful observation for highlighting the importance. The objective of this study was not to demonstrate direct the pathway environmental → food → human transmission, but rather to evaluate whether coordinated patterns of merA prevalence across environmental, food, and human compartments are compatible with ecological linkage among these matrices.

In the present study, parallel regional differences in merA prevalence were observed across environmental water, retail chicken meat, and human fecal samples. These coordinated distribution patterns suggest the presence of interconnected reservoirs operating within similar ecological context. However, the findings are interpreted as evidence of population level association rather than confirmation of directional transmission pathways. Importantly, the primary objective of this work is the comparative assessment of merA prevalence across regions and ecological compartments, thereby generating hypotheses regarding potential environmental–food–human linkages that needing further study or sourcing trace investigation.

**Comment 15**: *Why was shotgun metagenomic sequencing not performed to validate primer specificity and assess overall merA diversity?*

►**Response**:

We appreciate this valuable suggestion. Shotgun metagenomic sequencing is a powerful tool for characterizing microbial communities and *merA* diversity. However, the main aim of this study was not comprehensive analysis of *mer* operon diversity, but comparative quantification of the *merA* gene across different sample types and geographic regions using a rapid, scalable method.

For this purpose, targeted real-time PCR was selected because it provides sensitive, quantitative, and reproducible detection across a large number of heterogeneous samples, which is essential for cross-regional comparisons. In contrast, shotgun metagenomics can be influenced by sequencing depth, host DNA contamination, and bioinformatic variability, making quantitative comparisons across feces, food, and water samples more challenging.

**Comment 16**: *On what basis do you generalize horizontal gene transfer potential from sequencing a single Citrobacter freundii isolate?*

►**Response:**

We appreciate the Editor’s careful consideration of this point. Our inference regarding horizontal gene transfer potential is based on the genomic context identified in *C. freundii* TB25-009W-60-9. Whole-genome sequencing showed that *merA* is located on an IncFIB plasmid and flanked by insertion sequences consistent with mobile genetic elements. This plasmid-associated structure is widely recognized as being compatible with horizontal mobility among Enterobacteriaceae.

Thus, our conclusion is limited to showing that this representative isolate carries a genetic arrangement consistent with potential horizontal dissemination. We do not claim direct evidence of active transfer from a single isolate. Demonstration of actual transfer frequency and broader dissemination would require analysis of additional isolates and functional assays in future studies.

**Comment 17**: *Why were plasmid conjugation or transfer experiments not conducted to experimentally confirm horizontal transfer capability?*

►**Response**:

We thank the Editor for this insightful comment. The objective of the present study was to investigate the **distribution of the** merA **gene across environmental, food, and human-associated samples and to characterize its genomic context**, rather than to experimentally evaluate transfer mechanisms. In addition, whole-genome sequencing revealed that merA was located on an IncFIB plasmid and flanked by insertion sequences, genetic features widely recognized as indicators of mobile genetic elements with potential for horizontal dissemination.

In genomic epidemiology studies context, such structural evidence is commonly used to infer mobility potential and ecological dissemination capacity without requiring experimental transfer assays. However, the primary aim of this work was comparative prevalence analysis supported by genomic characterization, conjugation experiments fall outside the mechanistic scope of the current study.

**Comment 18**: *How might environmental factors (e.g., pH, sediment mercury, proximity to mining, industrial discharge influence merA prevalence, and why were these not assessed?*

►**Response**:

We thank the Editor for highlighting the potential influence of environmental variables on the distribution of merA. The primary aim of the present study, however, was to perform a **comparative cross-matrix and cross-regional assessment of** merA **prevalence using a standardized molecular approach**, rather than to identify site specific environmental drivers. Therefore, sampling was designed to capture representative community and environmental interfaces across regions, enabling comparison of biological indicators under real-world conditions. The interpretation of geographic differences was therefore contextualized using previously published environmental studies documenting regional mercury exposure sources, including artisanal gold mining activities in Ghana and industrial emissions in Vietnam.

Factors such as physicochemical conditions, sediment-associated mercury, and proximity to anthropogenic sources are indeed known to shape microbial community structure and mercury-resistance gene prevalence which measurements represent a complementary line of investigation distinct from the surveillance-oriented objectives of this study.

**Comment 19**: *Did you analyse potential co-selection between mercury resistant genes and antibiotic resistance genes in your plasmid or metagenomic data?*

►**Response**:

We thank the Editor for this important comment regarding the potential co-selection of heavy metal resistance determinants and antibiotic resistance genes. The primary focus of this study was to characterize the distribution and genomic context of *merA* across environmental, food, and human-associated compartments. Whole-genome sequencing of a representative *Citrobacter freundii* isolate was performed to determine the localization and structure of the *mer* operon and its associated mobile elements. Although potential co-selection between mercury resistance genes and antibiotic resistance genes was not directly examined in this study, the plasmid-borne localization of *merA* suggests a genomic context in which such co-selection may occur. This possibility warrants further investigation.

**Comment 20**: *Given the absence of direct human mercury exposure measurements, how do you justify the conclusion that fecal merA analysis can serve as a proxy for mercury-related public health risk?*

►**Response**:

We thank the Editor for this critical comment regarding interpretation of fecal *merA* detection in relation to mercury exposure and public health implications. Our intention was not to position fecal *merA* as a validated clinical biomarker of individual mercury exposure or as a direct measure of public health risk. Rather, the study aimed to explore whether microbial genetic indicators observed at the population level reflect broader environmental contexts in which mercury exposure may occur. This interpretation relates to population-level environmental context rather than individual toxicological burden.

**Comment 21**: *Provide the proper citation in material and methods- Add below appropriate reference-*

►**Response**:

Thank you for your helpful comment. We carefully reviewed the recommended references and incorporated those most relevant to the methodological aspects of the present study. Appropriate citations have been added in the Introduction and Discussion sections, and the corresponding text has been revised to ensure clarity and relevance.

**II. Response to Reviewer 4**

**Comment 1**: *The use of term “Multinational” in the title is misleading, as the study includes samples from only three countries. The title should be revised to more accurately reflect the geographic scope of the investigation.*

►**Response**:

We thank to Reviewer for the valuable comment. We have revised the title to: “Comparative prevalence of the mercury resistance gene *merA* in human feces, food, and environmental water from Japan, Vietnam, and Ghana”.

**Comment 2**: *The short title suggests that merA is being proposed as a biomarker for mercury contamination. However, merA is already well establish as a central gene in the mer operon, and this study does not demonstrate novelty in that regard. The title and conclusions should instead emphasize screening, distribution, or comparative prevalence of merA across different matrices and regions.*

►**Response**:

We sincerely thank the Reviewer for this insightful comment regarding the interpretation of the short title and the positioning of *merA* in the present study. The *merA* gene is widely recognized as a key component of the *mer* operon. Our objective in this work was not to propose *merA* as a novel biomarker, but rather to assess its distribution and detection frequency across diverse biological and environmental matrices. Accordingly, we have revised the short title as suggested.

**Comment 3**: *The manuscript contains repetitive and inconsistent phrasing (e.g., “Mercury (Hg) is a widespread environmental pollutant” followed by “Mercury is a toxic environmental pollutant”. Redundancy should be avoided. Abbreviations should be used consistently. Once introduced the abbreviation should be used throughout the manuscript.*

►**Response**:

We thank to Reviewer for identifying this important point. We have thoroughly revised of the entire manuscript to address these issues.

**Comment 4**: *In the introduction section, the authors state that methylmercury is the most toxic form of mercury affecting humans; however, the study focuses exclusively on merA, which is involved in the reduction of Hg(II) and does not play a role in methyl mercury detoxification. The introduction is poorly written and lacks logical flow. No clear detoxification pathway is described, and the role of merA within the mer operon, including its functional important, is not adequately explained.*

►**Response**:

We thank to Reviewer for this constructive comment. We appreciate the need to improve clarity and logical flow. We have revised the Introduction to provide a concise description of the *mer* operon structure and function, and explain the rationale for selecting *merA* as a molecular marker of bacterial mercury resistance and environmental mercury selection pressure.

**Comment 5**: *The manuscript does not identify the original source of mercury contamination in the three countries studied.*

►**Response**:

We thank to Reviewer for this important comment. The primary objective of this study was to compare the distribution of the mercury resistance gene *merA* across environmental, food, and human-associated samples, rather than to conduct a comprehensive source attribution analysis of mercury contamination in each country.

Nevertheless, we agree that contextual information regarding regional mercury inputs strengthens interpretation. In the Discussion section, we already summarize documented sources of mercury contamination in the study regions based on published literature. Artisanal and small-scale gold mining has been widely recognized as a major contributor to mercury release in Ghana, while industrial emissions and coal combustion have been reported as important sources in Vietnam. In contrast, Japan has implemented comparatively stringent environmental regulations following historical mercury pollution events. These contextual considerations are addressed with supporting references in the Discussion section

Overall, we emphasize that the conclusions of the present study are based on the comparison of merA prevalence across sample types and regions, interpreted in the context of previously reported environmental mercury data

**Comment 6**: *The overall study design is vague. The rationale for selecting specific sample sources (human feces, food, environmental water) is not clearly explained. It is unclear whether the sampled populations were exposed to mercury through environmental contamination, dietary intake, proximity to polluted water bodies, or other sources. For environmental water samples, the authors should clarify whether samples were filtered prior to analysis and whether turbidity, dust, or sand particles were present.*

►**Response**:

We thank to Reviewer for this important comment. The selection of environmental water, retail chicken meat, and human fecal samples was based on their roles as interconnected reservoirs, allowing for an investigation of mercury resistance determinants interconnected ecological compartments. Environmental water represents a potential environmental reservoir reflecting mercury related selective pressure. Retail chicken meat was included as a commonly consumed food matrix that may be influenced by environmental contamination or agricultural water use. Human fecal samples were analyzed to explore the presence of *merA* within the gut microbiome, which may reflect microbial exposure through environmental and dietary pathways. The aim of the study was therefore to compare the prevalence of *merA* across these matrices rather than to determine specific individual exposure routes such as proximity to contaminated sites or dietary mercury intake.

Regarding environmental water processing, samples were not membrane-filtered; instead, coarse debris and large particles were first removed by low-speed centrifugation (211 ×g, 1 min), followed by high-speed centrifugation (15,000 ×g, 5 min) to pellet microbial biomass for DNA extraction as stated in the Materials and Methods section. Although minor turbidity was present in some canal samples, visible sand and large particulate matter were effectively removed during the initial centrifugation step.

**Comment 7:** *The basis for selecting 60 μM HgCl₂ for bacterial isolation is not provided. The authors should justify this concentration with references or preliminary data.*

►**Response**:

We thank the Reviewer for this valuable comment and for requesting clarification regarding the HgCl₂ concentration used for bacterial isolation. The mercury concentration used in the selective medium for the isolation of mercury-resistant bacteria was selected based on previous reports and our preliminary experiments. We have now added this explanation, along with the appropriate reference, to the revised manuscript.

**Comment 8**: *The source of Citrobacter freundii strain TB25-009W-60-9 is unclear.*

►**Response**:

We thank the Reviewer for pointing out that the source of *Citrobacter freundii* strain TB25-009W-60-9 was unclear. We have now added a description of the strain and its origin in the revised manuscript.

**Comment 9**: *Given the extensive literature on the mer operon, the rationale for performing additional isolation and genomic analysis of mercury-resistant strains should be justified.*

►**Response**:

We thank the Reviewer for highlighting this important point. The source of *Citrobacter freundii* strain TB25-009W-60-9 has been clarified in Table 5 and our response to Comment 8.

The rationale for selecting this strain for whole-genome sequencing was not to redefine the structure or function of the *mer* operon, which has been extensively characterized in previous studies. Rather, the objective was to provide ecological and genomic context for mercury resistance within the environmental matrices investigated in the present study. Sequencing of a representative mercury-resistant isolate allowed us to confirm that the detected *merA* gene corresponded to a functional resistance determinant present in viable bacteria, and to examine the genomic location and surrounding genetic context of the *mer* operon within a strain recovered from the sampled environment. We acknowledge that analysis of a single isolate does not capture the full diversity of mercury resistance determinants present across these environments. Future work will therefore include whole-genome sequencing and comparative genomic analysis of additional mercury-resistant isolates to better characterize the diversity, mobility, and ecological distribution of *mer*-associated genetic elements across different sample types and geographic regions.

**Comment 10:** *Several subsection headings in the Results section resemble those of the Materials and Methods section and should be revise to reflect actual findings rather than procedures.*

►**Response**:

We thank the Reviewer for this helpful suggestion regarding the organization of the Results section.

According to your suggestion, the subsection headings in the Results section have been revised to better reflect the main observations and outcomes of the analyses.

**Comment 11**: *The authors should clarify whether the mercury detected in human fecal samples is more likely linked to dietary sources (e.g., chicken consumption) or drinking water contamination.*

►**Response**:

We thank the Reviewer for raising this important point regarding potential exposure pathways contributing to the presence of *merA* in human fecal samples. The present study was designed to compare the prevalence of mercury resistance genes across environmental, food, and human-associated matrices, rather than to determine specific exposure routes at the individual level.

Both dietary intake and environmental water exposure represent biological pathways through which mercury associated microbial populations may influence the human gut microbiome. However, because individual dietary habits, drinking water sources, and personal exposure histories were not assessed, the current dataset does not allow attribution of fecal *merA* detection to a single dominant exposure source. Instead, the study evaluates whether concordant patterns of *merA* occurrence across environmental water, food samples, and human feces are consistent with shared environmental exposure contexts at a population level.

**Comment 12**: *I do not find the figures in this version of ms.*

►**Response**:

The submission guidelines do not require figures to be inserted into the main manuscript file. According to the PLOS ONE guidelines, figures should not be included in the text file, but instead uploaded separately as individual files.

**Comment 13**: *The isolation of mercury-resistant bacteria and genomic analysis of a single strain are not well aligned with the stated objectives in the Introduction.*

►**Response**:

We thank the Reviewer highlighting the need to clarify the relationship between the study objectives and the isolation and genomic characterization of a mercury-resistant strain. The primary objective of this study is the comparative assessment of merA prevalence across environmental, food, and human-associated matrices. The isolation and genomic analysis of a representative mercury-resistant bacterium were included to provide **biological and genomic context** for the molecular detection results. Specifically, characterization of an isolate carrying the mer operon allows confirmation that the detected merA signals correspond to functional mercury resistance systems present in viable bacteria and enables examination of the genetic environment associated with mercury resistance within the studied ecosystem. Thus, the genomic analysis in this study was intended as a complementary component linking gene-based surveillance to organism level characterization, rather than as a separate mechanistic investigation.

**Comment 14**: *The authors should either strengthen the rationale linking these analyses to the study goals or revise the scope of the manuscript accordingly.*

►**Response:**

We thank the Reviewer for this important recommendation regarding conceptual alignment between the study objectives and the analyses performed. The primary objective of this study was to investigate the distribution and prevalence of the mercury resistance gene ***merA*** across environmental, food, and human-associated samples in different geographic regions.

To support this objective, several complementary analyses were included. Quantification of ***merA*** allowed comparison of gene prevalence across sample matrices and regions. Measurement of mercury concentrations in environmental water samples provided the information regarding potential environmental selection pressure. In addition, isolation and characterization of mercury-resistant bacteria enabled confirmation that viable bacteria carrying mercury resistance determinants were present in the sampled environments. Genome sequencing of the representative isolate further provided genetic evidence of mercury resistance mechanisms. Together, these analyses provide multiple lines of evidence supporting the interpretation of ***merA* distribution across ecological compartments**.
