# Supplementary material for: Comparative prevalence of the mercury resistance gene merA in human feces, food, and environmental water from Japan, Vietnam, and Ghana
Source: PLoS One. 2026 Sep 8;21(9):e0357976. doi: 10.1371/journal.pone.0357976 (PMC13552768; doi:10.1371/journal.pone.0357976)
Supplement: Supplementary file 7 [file pone.0357976.s011.docx]

**PLOS ONE**

**PONE-D-25-30013R3**

Manuscript title: “Comparative prevalence of the mercury resistance gene *merA* in human feces, food, and environmental water from Japan, Vietnam, and Ghana”.

We sincerely thank Reviewer for your through and this careful evaluation of our manuscript. Your insightful comments and questions have helped us significantly improve the clarity of our work. We have revised the manuscript extensively in response to all suggestions.

Below we provide a point-by-point response. All changes have been incorporated into the revised manuscript and are indicated accordingly.

**Response to Reviewer #5**

**Comment 1**:

Line 56 in the introduction is redundant and may be removed as the same information is conveyed in other sentences. Hg(II) is never mentioned clearly as the most common and/or toxic form of environmental mercury, which may be important as the study revolves around *merA*, an Hg(II) reducing gene.

►**Response:**

We thank to Reviewer for the valuable observation. We have removed the redundant sentence from line 56 and added a brief explanation of Hg(II)(**line 56-63**), as suggested.

**Comment 2**:

Line 72 may include a sentence about any previous literature indicating the prevalence of HGT of mercury resistance or even heavy metal resistance genes in aquatic ecosystems, since it is relevant to the genome analysis performed in the study.

►**Response:**

We thank to Reviewer for this constructive suggestion. We have added a sentence in the Introduction section (**line 83-86**) referencing previous studies in horizontal gene transfer of mercury resistance genes in aquatic environments which provide context for our genomic analysis of the *mer* operon localization plasmid.

**Comment 3**:

In the line 145 of the methods section, it is more meaningful to include the concentration rather than the volume of DNA obtained. In the results for the abundance of *merA,* the quantification results would have benefited from the use of a “control” gene, such as the 16S, in order to properly assess the microbial population abundance in the samples. The authors have mentioned that they extracted DNA from the same amount of starting material, but were the final DNA concentrations comparable between samples after extraction, or was any kind of normalization performed between samples for better interpretation of *merA* abundance?

►**Response**:

We thank to Reviewer for identifying these important points.

Regarding normalization and the use of a control gene, we appreciate this valuable methodological suggestion. We agree that normalization to a housekeeping gene such as the 16S rRNA gene would provide a more refined assessment of *merA* abundance relative to the total bacterial load. However, our primary aim was to compare *merA* detection rates and relative copy numbers under consistent experimental conditions rather than to determine absolute *merA* abundance. In accordance with the reviewer's suggestion, we have added the range of DNA concentrations obtained from the extracted samples to the Materials and methods section (**line 168-171**). We have also revised the Discussion to include a description of the limitations of this method (**line 366-375**).

**Comment 4**:

The lack of mercury measurements from the stool and meat samples, which will help correlate the Hg prevalence and *merA* abundance, is never addressed, especially since mercury is bioaccumulated across the trophic levels.

Were any sequence similarity or phylogenetic analyses performed for the detected IncFIB plasmid to infer the occurrence of an HGT event?

►**Response**:

We thank to Reviewer for the insightful comment.

Regarding mercury measurements in stool and meat samples, the measuring total mercury concentrations in stool and meat samples would have provided valuable complementary data to correlate with *merA* abundance. However, the primary objective of this study was to investigate the prevalence of the *merA* gene as a biomarker of mercury resistance, rather than to directly quantify mercury levels in biological matrices. While mercury bioaccumulation across trophic levels is well documented, the relationship between mercury concentrations and *merA* prevalence is complex, as *merA* reflects the presence of mercury-resistant bacteria rather than directly measuring mercury content. We have added a discussion of this limitation and the need for future studies incorporating direct mercury measurements in the biological samples to the Limitations section (**line 408-420**).

Regarding phylogenetic analysis of the IncFIB plasmid, we performed comparative genomic analysis of the IncFIB plasmid from C*. freundii* TB25-009W-60-9 against the reference plasmid from *Klebsiella pneumonia* 1_GR_13. The analysis revealed that *mer* operon structure was nearly identical between the two plasmids, while most other genetic regions showed no homology (Fig S2). This finding suggests that *mer* operon may have been acquired by the IncFIB plasmid through HGT events. Additionally, the *mer* operon in our isolate was flanked by two insertion sequences (ISs), which further supports the potential for horizontal transfer. We have expanded the Discussion section (**line 384-391**) to include these observations and their implication for HGT.
