# Supplementary material for: Comparative prevalence of the mercury resistance gene merA in human feces, food, and environmental water from Japan, Vietnam, and Ghana
Source: PLoS One. 2026 Sep 8;21(9):e0357976. doi: 10.1371/journal.pone.0357976 (PMC13552768; doi:10.1371/journal.pone.0357976)
Supplement: Peer review history

# Peer review: Decision Letter 0

**DOI:** 10.1371/journal.pone.0357976.r001

**Authors:** Naser Anjum

2 Aug 2025

Dear Dr. Yamamoto,

Thank you for submitting your manuscript to PLOS ONE. After careful consideration, we feel that it has merit but does not fully meet PLOS ONE’s publication criteria as it currently stands. Therefore, we invite you to submit a revised version of the manuscript that addresses the points raised during the review process.

Please submit your revised manuscript by Sep 16 2025 11:59PM. If you will need more time than this to complete your revisions, please reply to this message or contact the journal office at plosone@plos.org. When you're ready to submit your revision, log on to https://www.editorialmanager.com/pone/ and select the 'Submissions Needing Revision' folder to locate your manuscript file.

- A rebuttal letter that responds to each point raised by the academic editor and reviewer(s). You should upload this letter as a separate file labeled 'Response to Reviewers'.
- A marked-up copy of your manuscript that highlights changes made to the original version. You should upload this as a separate file labeled 'Revised Manuscript with Track Changes'.
- An unmarked version of your revised paper without tracked changes. You should upload this as a separate file labeled 'Manuscript'.

If applicable, we recommend that you deposit your laboratory protocols in protocols.io to enhance the reproducibility of your results. Protocols.io assigns your protocol its own identifier (DOI) so that it can be cited independently in the future. For instructions see: https://journals.plos.org/plosone/s/submission-guidelines#loc-laboratory-protocols. Additionally, PLOS ONE offers an option for publishing peer-reviewed Lab Protocol articles, which describe protocols hosted on protocols.io. Read more information on sharing protocols at https://plos.org/protocols?utm_medium=editorial-email&utm_source=authorletters&utm_campaign=protocols.

We look forward to receiving your revised manuscript.

Kind regards,

Naser A. Anjum, PhD

Academic Editor

PLOS ONE

Journal Requirements:

When submitting your revision, we need you to address these additional requirements.

1. Please ensure that your manuscript meets PLOS ONE's style requirements, including those for file naming. The PLOS ONE style templates can be found at https://journals.plos.org/plosone/s/file?id=wjVg/PLOSOne_formatting_sample_main_body.pdf and https://journals.plos.org/plosone/s/file?id=ba62/PLOSOne_formatting_sample_title_authors_affiliations.pdf

2. Please include a complete copy of PLOS’ questionnaire on inclusivity in global research in your revised manuscript. Our policy for research in this area aims to improve transparency in the reporting of research performed outside of researchers’ own country or community. The policy applies to researchers who have travelled to a different country to conduct research, research with Indigenous populations or their lands, and research on cultural artefacts. The questionnaire can also be requested at the journal’s discretion for any other submissions, even if these conditions are not met. Please find more information on the policy and a link to download a blank copy of the questionnaire here: https://journals.plos.org/plosone/s/best-practices-in-research-reporting. Please upload a completed version of your questionnaire as Supporting Information when you resubmit your manuscript.

3. In your Methods section, please provide additional information regarding the permits you obtained for the work. Please ensure you have included the full name of the authority that approved the field site access and, if no permits were required, a brief statement explaining why.

4. Thank you for stating the following financial disclosure:

This work was supported by the Japan Society for the Promotion of Science (Grant no. 23H00446).

Please state what role the funders took in the study. If the funders had no role, please state: "The funders had no role in study design, data collection and analysis, decision to publish, or preparation of the manuscript."

If this statement is not correct you must amend it as needed.

Please include this amended Role of Funder statement in your cover letter; we will change the online submission form on your behalf.

5. Please note that funding information should not appear in any section or other areas of your manuscript. We will only publish funding information present in the Funding Statement section of the online submission form. Please remove any funding-related text from the manuscript.

6. Please include a separate caption for each figure in your manuscript.

7. Please remove all personal information, ensure that the data shared are in accordance with participant consent, and re-upload a fully anonymized data set.

Note: spreadsheet columns with personal information must be removed and not hidden as all hidden columns will appear in the published file.

Additional guidance on preparing raw data for publication can be found in our Data Policy (https://journals.plos.org/plosone/s/data-availability#loc-human-research-participant-data-and-other-sensitive-data) and in the following article: http://www.bmj.com/content/340/bmj.c181.long.

8. If the reviewer comments include a recommendation to cite specific previously published works, please review and evaluate these publications to determine whether they are relevant and should be cited. There is no requirement to cite these works unless the editor has indicated otherwise.

9. Please review your reference list to ensure that it is complete and correct. If you have cited papers that have been retracted, please include the rationale for doing so in the manuscript text, or remove these references and replace them with relevant current references. Any changes to the reference list should be mentioned in the rebuttal letter that accompanies your revised manuscript. If you need to cite a retracted article, indicate the article’s retracted status in the References list and also include a citation and full reference for the retraction notice.

Additional Editor Comments :

REVIEWER # 1

The authors attempt to the prevalence and abundance of the mercury resistance gene merA in human feces, retail chicken meat, and environmental water samples collected from Japan, Vietnam, and Ghana.

The work is important because the main focus is on the mercury resistance gene merA as a biomarker for regional environmental mercury contamination. They investigated the prevalence and abundance of merA in fecal samples from local residents, food, and environmental water across multiple countries. Furthermore, they evaluated its potential utility as an indicator of Hg contamination.

The transfer of genes heavy metal between microbial species can not only increase the prevalence of resistance but also increase the pathogenicity and adaptability of microorganisms to hostile environments

The article is very simple and easy to understand

The manuscript is for the most part well written and shows robust methods, the abstract section is appropriate.

The references cited are the right ones.

The article is scientifically rigorous and the findings are thoroughly discussed.

REVIEWER # 2

Overall, the paper is well written, but I suggest the author emphasize the public health implications of mercury contamination more strongly in the introduction and discussion sections. My specific comments are as follows:

1. As the authors acknowledge that mercury is a naturally occurring substance, it would be beneficial to include information about the acceptable threshold levels that are not hazardous to human health, as well as the points at which these levels become harmful. Additionally, it's worth noting that the levels of mercury observed in Ghana and Vietnam exceed these thresholds.

2. There seems to be a slight variation in the prevalence of mercury in water compared to human and chicken samples across different countries. I would appreciate clarification on this discrepancy.in discussion

3. The methodology section lacks clarity regarding the specific experiments conducted for the various samples. It appears that culture techniques were only applied to water samples, but need a justification why it was not applied to other samples? A flow diagram would enhance understanding of the methodology used.

4. Statistical analysis is not explicitly explained whether it was bivariate or multivariate analysis? If the number of sample is less than 30, which statistical test was performed

5. In Table 5, some of the samples were not tested by any methods. Could the authors explain the reasons for this?

6. I recommend rewriting lines 189 to 194 for clarity. It would be helpful to specify which databases or software were used for annotation, the purpose of utilizing ResFinder (e.g., for investigating the merA gene in silico), and the reason for using Geneious Prime.

7. In line 5, please cite the reference using a numerical format rather than including a hyperlink.

8. There should be a consistent structure applied to all tables. Overall, the formatting quality of the tables is poor.

9. Figure 1 does not clearly indicate which dots correspond to which countries. Improvements in clarity would be beneficial.

[Note: HTML markup is below. Please do not edit.]

Reviewers' comments:

Reviewer's Responses to Questions

**Comments to the Author**

1. Is the manuscript technically sound, and do the data support the conclusions?

Reviewer #1: Yes

Reviewer #2: Yes

**********

2. Has the statistical analysis been performed appropriately and rigorously?

Reviewer #1: Yes

Reviewer #2: No

**********

3. Have the authors made all data underlying the findings in their manuscript fully available?

The PLOS Data policy

Reviewer #1: Yes

Reviewer #2: Yes

**********

4. Is the manuscript presented in an intelligible fashion and written in standard English?

Reviewer #1: Yes

Reviewer #2: Yes

**********

Reviewer #1: The authors attempt to the prevalence and abundance of the mercury resistance gene merA in human feces, retail chicken meat, and environmental water samples collected from Japan, Vietnam, and Ghana.

The work is important because the main focus is on the mercury resistance gene merA as a biomarker for regional environmental mercury contamination. They investigated the prevalence and abundance of merA in fecal samples from local residents, food, and environmental water across multiple countries. Furthermore, they evaluated its potential utility as an indicator of Hg contamination.

The transfer of genes heavy metal between microbial species can not only increase the prevalence of resistance but also increase the pathogenicity and adaptability of microorganisms to hostile environments

The article is very simple and easy to understand

The manuscript is for the most part well written and shows robust methods, the abstract section is appropriate.

The references cited are the right ones.

The article is scientifically rigorous and the findings are thoroughly discussed.

Reviewer #2: Overall, the paper is well written, but I suggest the author emphasize the public health implications of mercury contamination more strongly in the introduction and discussion sections. My specific comments are as follows:

1. As the authors acknowledge that mercury is a naturally occurring substance, it would be beneficial to include information about the acceptable threshold levels that are not hazardous to human health, as well as the points at which these levels become harmful. Additionally, it's worth noting that the levels of mercury observed in Ghana and Vietnam exceed these thresholds.

2. There seems to be a slight variation in the prevalence of mercury in water compared to human and chicken samples across different countries. I would appreciate clarification on this discrepancy.in discussion

3. The methodology section lacks clarity regarding the specific experiments conducted for the various samples. It appears that culture techniques were only applied to water samples, but need a justification why it was not applied to other samples? A flow diagram would enhance understanding of the methodology used.

4. Statistical analysis is not explicitly explained whether it was bivariate or multivariate analysis? If the number of sample is less than 30, which statistical test was performed

5. In Table 5, some of the samples were not tested by any methods. Could the authors explain the reasons for this?

6. I recommend rewriting lines 189 to 194 for clarity. It would be helpful to specify which databases or software were used for annotation, the purpose of utilizing ResFinder (e.g., for investigating the merA gene in silico), and the reason for using Geneious Prime.

7. In line 5, please cite the reference using a numerical format rather than including a hyperlink.

8. There should be a consistent structure applied to all tables. Overall, the formatting quality of the tables is poor.

9. Figure 1 does not clearly indicate which dots correspond to which countries. Improvements in clarity would be beneficial.

**********

what does this mean?). If published, this will include your full peer review and any attached files.

If you choose “no”, your identity will remain anonymous but your review may still be made public.

**Do you want your identity to be public for this peer review?**  For information about this choice, including consent withdrawal, please see our Privacy Policy

Reviewer #1: No

Reviewer #2: No

**********

[NOTE: If reviewer comments were submitted as an attachment file, they will be attached to this email and accessible via the submission site. Please log into your account, locate the manuscript record, and check for the action link "View Attachments". If this link does not appear, there are no attachment files.]

While revising your submission, please upload your figure files to the Preflight Analysis and Conversion Engine (PACE) digital diagnostic tool, https://pacev2.apexcovantage.com/. PACE helps ensure that figures meet PLOS requirements. To use PACE, you must first register as a user. Registration is free. Then, login and navigate to the UPLOAD tab, where you will find detailed instructions on how to use the tool. If you encounter any issues or have any questions when using PACE, please email PLOS at figures@plos.org

# Author comment: Author response to Decision Letter 1

**DOI:** 10.1371/journal.pone.0357976.r002

30 Sep 2025

Response to the comments

Journal requirements:

1. Please ensure that your manuscript meets PLOS ONE's style requirements, including those for file naming.

►Response:

Yes, we have checked that the file names you mentioned match the required style.

2. Please include a complete copy of PLOS’ questionnaire on inclusivity in global research in your revised manuscript.

►Response:

We have attached the questionnaire as per your suggestion.

3. In your Methods section, please provide additional information regarding the permits you obtained for the work.

►Response:

We added it as per your suggestion (Line 95-99, 102-106).

4. 5. Please state what role the funders took in the study.

►Response:

We removed the funding-related text from the paper and stated the role the funders took in the study in Financial Disclosure.

6. Please include a separate caption for each figure in your manuscript.

►Response:

Yes, we did as per your suggestion (Line 255-257, 290-292).

7. Please remove all personal information, ensure that the data shared are in accordance with participant consent.

►Response:

Yes, we have checked to make sure it does not contain any personal information.

8. If the reviewer comments include a recommendation to cite specific previously published works, please review and evaluate these publications to determine whether they are relevant and should be cited.

►Response:

Yes, we have confirmed this.

9. Please review your reference list to ensure that it is complete and correct.

►Response:

Yes, we have checked that the references are properly cited.

Reviewer #1

The authors attempt to the prevalence and abundance of the mercury resistance gene merA in human feces, retail chicken meat, and environmental water samples collected from Japan, Vietnam, and Ghana. The work is important because the main focus is on the mercury resistance gene merA as a biomarker for regional environmental mercury contamination. ----------------.

►Response:

We sincerely thank the reviewer for your positive and encouraging comments regarding our manuscript.

Reviewer #2:

Overall, the paper is well written, but I suggest the author emphasize the public health implications of mercury contamination more strongly in the introduction and discussion sections.

►Response:

We sincerely thank the reviewer for the constructive and thoughtful feedback provided. In accordance with the reviewer’s valuable feedback, we have included additional information in the Introduction on the potential health impacts of mercury contamination (Line 56-57).

Comment 1:

As authors acknowledge that mercury is a naturally occurring substance, it would be beneficial to include information about the acceptable threshold levels that are not hazardous to human health, as well as the points at which these levels become harmful. Additionally, it’s worth noting that the levels of mercury observed in Ghana and Vietnam exceed these thresholds.

►Response:

Thank you for this important suggestion. We appreciate your insightful comment regarding the need to include information on acceptable mercury threshold levels. As suggested, we have added information on acceptable threshold levels of mercury in water and compared them with the levels reported in Ghana and Vietnam. According to the WHO guidelines, the permissible limit of mercury in drinking water is 0.001 mg/L. The national regulation of both Vietnam and Ghana adopt the same threshold of 0.001 mg/L for drinking and surface water. We have now incorporated this information into the Introduction and Discussion to emphasize the public health significance of our findings (Line 64-71, 342-349).

Comment 2:

There seems to be a slight variation in the prevalence of mercury in water compared to human and chicken sample across different countries. I would appreciate clarification on this discrepancy in discussion.

►Response:

We thank the reviewer for this insightful comment. Indeed, the prevalence of merA in water did not always align with human stool or chicken samples across countries. We have now expanded our discussion to clarify this point in the revised manuscript as suggested by the reviewer (Line 322-331).

Comment 3:

Methodology section lacks clarify regarding which experiments were conducted for various samples. It appears that culture techniques were only applied to water samples, but need a justification why it was not applied to other samples? A flow diagram would enhance understanding of the methodology used.

►Response:

We sincerely thank the reviewer for this insightful comment. In accordance with the reviewers’ comments, we have clarified in the revised manuscript the rationale for using only environmental water in the culture experiments. We hope that this addition appropriately addresses the reviewer’s comment (Line 276-279).

The sample processing procedures used in this study are relatively simple, and we believe that the experiments can be readily reproduced based on the information provided in the Methods section. If the inclusion of a scheme diagram is deemed necessary, we would be happy to provide one upon your further instruction.

Comment 4:

Statistical analysis is not explicitly explained whether it was bivariate or multivariate analysis. If the number of samples is less than 30, which statistical test was performed.

►Response:

Thank you for pointing this out. Statistical analyses in our study were conducted as bivariate comparisons. We have clarified this point in the revised Statistical analysis subsection of the Materials and methods (Line 214-215).

Comment 5:

In Table 5, some samples were not tested any methods. Could the authors explain the reason for this?

►Response:

We appreciate the reviewer’s comment. All environmental water samples were first tested for the presence of the merA gene by real-time PCR. For subsequent culture work, we focused on a subset of merA-positive samples in order to obtain representative mercury-resistant bacterial isolates for species identification and genomic characterization. This approach was taken because the objective was not to culture every merA-positive sample, but rather to demonstrate the diversity of mercury-resistant bacteria present and to obtain representative strains for further genomic analysis (Line 276-279). Table 5 therefore shows only those isolates that were successfully cultured and identified.

Comment 6:

I recommend rewriting lines 189 to 194 to clarify. It would be helpful to specify which databases or software were used for annotation, the purpose of utilizing ResFinder (e.g., for investigating the merA gene in silico), and the reason for using Geneious Prime.

►Response:

We thank the reviewer for this valuable suggestion. We have revised the text to more clearly describe the genome annotation and analysis process. Specifically, we now state that genome annotation was performed using the DFAST tool, resistance genes were investigating with ResFinder, plasmid replicons were identified using PlasmidFinder, and Geneious Prime was employed for sequence visualization and comparative analysis of gene structure (Line 204-210).

Comment 7:

In line 55, please cite the reference using a numerical format rather than including a hyperlink.

►Response:

We thank the reviewer for pointing out the inconsistency. In line 55, we had included both numerical reference [2] and hyperlink, which referred to a different source. Since our intended citation is Reference [2] (Donkor et al., 2006), we have removed the hyperlink to ensure consistency with the numerical citation format required by the journal (Line 55).

Comment 8:

There should be a consistent structure applied to all tables. Overall, the formatting quality of the table is poor.

►Response:

We appreciate the reviewer’s suggestion. We have taken care to confirm that all tables in the manuscript adhere to the submission requirements and have made appropriate adjustments where necessary.

Comment 9:

Figure 1 does not clearly indicate which dots correspond to which countries. Improvements in clarity would be beneficial.

►Response:

The incorrect positioning of the country names in Figure 1 seems to have been caused by the PDF conversion process. In the revised version, we will double-check to ensure all labels are displayed correctly. Thank you very much for your helpful comment.

Attachment Submitted filename: Response to reviewers-PONE-D-25-30012 R.docx

# Peer review: Decision Letter 1

**DOI:** 10.1371/journal.pone.0357976.r003

**Authors:** Rajesh Singh Rathore

13 Jan 2026

Dear Dr. Yamamoto,

I hope this email finds you well.

Thank you very much for your careful handling of our manuscript entitled **“Multinational comparison of the prevalence of the mercury resistance biomarker gene**  ***merA***  **in human feces, food, and environmental water.”**  We are grateful to you and the reviewers for the constructive comments and for the recommendation of **minor revision** .

We have now revised the manuscript accordingly, addressing all reviewer comments in detail. The revisions include clarification of methodological aspects, improvements in statistical descriptions, enhanced discussion of mercury threshold levels and public health implications, and corrections to formatting, figures, tables, and referencing style. A point-by-point response to the reviewers’ comments has been provided to clearly indicate how each concern was addressed.

We sincerely appreciate the time and effort invested by the reviewers and the editorial team, which have greatly helped improve the quality and clarity of our manuscript. We hope that the revised version is now suitable for publication.

Thank you again for your consideration. Please do not hesitate to contact us if any further information is required.

With kind regards,

Rajesh

Please submit your revised manuscript by Feb 27 2026 11:59PM. If you will need more time than this to complete your revisions, please reply to this message or contact the journal office at plosone@plos.org. When you're ready to submit your revision, log on to https://www.editorialmanager.com/pone/ and select the 'Submissions Needing Revision' folder to locate your manuscript file.

Please include the following items when submitting your revised manuscript:

- A letter that responds to each point raised by the academic editor and reviewer(s). You should upload this letter as a separate file labeled 'Response to Reviewers'.
- A marked-up copy of your manuscript that highlights changes made to the original version. You should upload this as a separate file labeled 'Revised Manuscript with Track Changes'.
- An unmarked version of your revised paper without tracked changes. You should upload this as a separate file labeled 'Manuscript'.

If applicable, we recommend that you deposit your laboratory protocols in protocols.io to enhance the reproducibility of your results. Protocols.io assigns your protocol its own identifier (DOI) so that it can be cited independently in the future. For instructions see: https://journals.plos.org/plosone/s/submission-guidelines#loc-laboratory-protocols. Additionally, PLOS ONE offers an option for publishing peer-reviewed Lab Protocol articles, which describe protocols hosted on protocols.io. Read more information on sharing protocols at https://plos.org/protocols?utm_medium=editorial-email&utm_source=authorletters&utm_campaign=protocols.

We look forward to receiving your revised manuscript.

Kind regards,

Rajesh Singh Rathore, Ph.D

Academic Editor

PLOS One

Journal Requirements:

If the reviewer comments include a recommendation to cite specific previously published works, please review and evaluate these publications to determine whether they are relevant and should be cited. There is no requirement to cite these works unless the editor has indicated otherwise.

Please review your reference list to ensure that it is complete and correct. If you have cited papers that have been retracted, please include the rationale for doing so in the manuscript text, or remove these references and replace them with relevant current references. Any changes to the reference list should be mentioned in the rebuttal letter that accompanies your revised manuscript. If you need to cite a retracted article, indicate the article’s retracted status in the References list and also include a citation and full reference for the retraction notice.

[Note: HTML markup is below. Please do not edit.]

Reviewers' comments:

Reviewer's Responses to Questions

**Comments to the Author**

Reviewer #1: All comments have been addressed

Reviewer #3: All comments have been addressed

**********

2. Is the manuscript technically sound, and do the data support the conclusions?

Reviewer #1: Yes

Reviewer #3: Yes

**********

3. Has the statistical analysis been performed appropriately and rigorously?

Reviewer #1: Yes

Reviewer #3: Yes

**********

4. Have the authors made all data underlying the findings in their manuscript fully available?

The PLOS Data policy

Reviewer #1: Yes

Reviewer #3: Yes

**********

5. Is the manuscript presented in an intelligible fashion and written in standard English?

Reviewer #1: Yes

Reviewer #3: Yes

**********

Reviewer #1: The authors have responded point by point to the reviewers' recommendations and have done a good job.

Reviewer #3: The authors investigated the prevalence and abundance of the mercury resistance gene merA in human feces, retail chicken meat, and environmental water samples collected from Japan, Vietnam, and Ghana. Although the current approach is not exhaustive, it provides meaningful insights into the geographical distribution and prevalence of merA-harboring bacteria. The revised version manuscript was evaluated thoroughly, and while the draft has been significantly improved, there are minor concerns related to formatting. Please find my comments below:

Comments:

1. Line 66, 67, 68: Please cite in proper referencing format rather than links.

2. Please confirm referencing style in both figure captions as well as in body.

3. Line 149, 174, 204,206,207: Please use proper citation style.

4. Line 227: It should be Fig. S1 and not S1 Fig (Please check journal format if needed). Same comment for Line 222, 227,288.

**********

what does this mean?). If published, this will include your full peer review and any attached files.

If you choose “no”, your identity will remain anonymous but your review may still be made public.

**Do you want your identity to be public for this peer review?**  For information about this choice, including consent withdrawal, please see our Privacy Policy

Reviewer #1: No

Reviewer #3: No

**********

[NOTE: If reviewer comments were submitted as an attachment file, they will be attached to this email and accessible via the submission site. Please log into your account, locate the manuscript record, and check for the action link "View Attachments". If this link does not appear, there are no attachment files.]

To ensure your figures meet our technical requirements, please review our figure guidelines: https://journals.plos.org/plosone/s/figures

You may also use PLOS’s free figure tool, NAAS, to help you prepare publication quality figures: https://journals.plos.org/plosone/s/figures#loc-tools-for-figure-preparation.

NAAS will assess whether your figures meet our technical requirements by comparing each figure against our figure specifications.

Attachment Submitted filename: Comments_to_the_authors.docx

# Author comment: Author response to Decision Letter 2

**DOI:** 10.1371/journal.pone.0357976.r004

29 Jan 2026

Response to the comments

1. Line 66, 67, 68: Please cite in proper referencing format rather than links.

►Response: As suggested, we have reformatted the cited web addresses according to the PLOS ONE reference style.

2. Please confirm referencing style in both figure captions as well as in body.

►Response: In accordance with the reviewer’s suggestion, we have unified the citation style throughout the manuscript.

3. Line 149, 174, 204,206,207: Please use proper citation style.

►Response: we have reformatted the cited web addresses according to the PLOS ONE reference style.

4. Line 227: It should be Fig. S1 and not S1 Fig (Please check journal format if needed). Same comment for Line 222, 227,288.

►Response: In accordance with the reviewer’s comment, we revised “S1 Fig” to “Fig. S1” and made the same correction throughout the manuscript.

Attachment Submitted filename: Response to reviewers-PONE-D-25-30012 (002).docx

# Peer review: Decision Letter 2

**DOI:** 10.1371/journal.pone.0357976.r005

**Authors:** Rajesh Singh Rathore

19 Feb 2026

Dear Dr. Yamamoto,

Thank you for submitting your manuscript entitled *“Multinational Comparison of the Prevalence of the Mercury Resistance Biomarker Gene merA in Human Feces, Food, and Environmental Water.”*

Following peer review, the manuscript requires **major revision**  before further consideration. While the study is timely and valuable, the reviewers have raised significant concerns regarding methodological clarity, data interpretation, validation of merA as a biomarker, and the strength of the public health conclusions.

Please carefully address all reviewer comments and submit:

1. A revised manuscript (tracked and clean versions), and
2. A detailed point-by-point response to each comment.

We look forward to receiving your revised submission.

Sincerely,

Rajesh

Please submit your revised manuscript by Apr 05 2026 11:59PM. If you will need more time than this to complete your revisions, please reply to this message or contact the journal office at plosone@plos.org. When you're ready to submit your revision, log on to https://www.editorialmanager.com/pone/ and select the 'Submissions Needing Revision' folder to locate your manuscript file.

Please include the following items when submitting your revised manuscript:

- A letter that responds to each point raised by the academic editor and reviewer(s). You should upload this letter as a separate file labeled 'Response to Reviewers'.
- A marked-up copy of your manuscript that highlights changes made to the original version. You should upload this as a separate file labeled 'Revised Manuscript with Track Changes'.
- An unmarked version of your revised paper without tracked changes. You should upload this as a separate file labeled 'Manuscript'.

If you would like to make changes to your financial disclosure, please include your updated statement in your cover letter. Guidelines for resubmitting your figure files are available below the reviewer comments at the end of this letter.

If applicable, we recommend that you deposit your laboratory protocols in protocols.io to enhance the reproducibility of your results. Protocols.io assigns your protocol its own identifier (DOI) so that it can be cited independently in the future. For instructions see: https://journals.plos.org/plosone/s/submission-guidelines#loc-laboratory-protocols. Additionally, PLOS ONE offers an option for publishing peer-reviewed Lab Protocol articles, which describe protocols hosted on protocols.io. Read more information on sharing protocols at https://plos.org/protocols?utm_medium=editorial-email&utm_source=authorletters&utm_campaign=protocols.

We look forward to receiving your revised manuscript.

Kind regards,

Rajesh Singh Rathore, Ph.D

Academic Editor

PLOS One

Journal Requirements:

If the reviewer comments include a recommendation to cite specific previously published works, please review and evaluate these publications to determine whether they are relevant and should be cited. There is no requirement to cite these works unless the editor has indicated otherwise.

Additional Editor Comments:

Comments -

1. Can you provide statistical correlation analysis between merA copy numbers and measured mercury concentrations in water samples to support your biomarker claim?

2. Why was mercury speciation (e.g., methylmercury vs. inorganic mercury) not performed, given that MeHg is the most toxic and biologically relevant form?

3. Why were mercury concentrations not measured in Ghanaian environmental water samples, and how can geographic comparisons be made without these data?

4. If fecal merA is proposed as a proxy for human mercury exposure, why were mercury levels not directly measured in human feces, blood, hair, or urine samples?

5. Why were mercury concentrations not determined in chicken meat samples to validate the proposed food-chain transmission hypothesis?

6. How do you account for the relatively small and uneven sample sizes across countries (e.g., Ghana chicken n=10)? Was a power calculation performed?

7. Why were merA copy numbers not normalized to total bacterial abundance (e.g., 16S rRNA gene copies) to control for differences in microbial biomass?

8. How do you justify the use of E. coli genomic DNA as a universal standard for merA quantification, given variability in genome size and plasmid copy number among species?

9. Why were Cq values outside the validated standard curve range extrapolated, and how might this affect quantitative accuracy?

10. Given that at least seven merA determinants exist, how do you address potential underestimation due to using only a single primer–probe set?

11. Does detection of merA DNA confirm functional resistance? Have you performed RT-qPCR or phenotypic assays to confirm gene expression or enzymatic activity?

12. Why were potential confounding variables in human participants (dietary habits, fish consumption, occupational exposure, antibiotic use, age, sex) not analyzed?

13. How do you explain the high prevalence of merA in Japanese environmental water despite low detection in human feces and chicken meat?

14. Can you clarify how a cross-sectional study design allows inference regarding transmission pathways (environment → food → human)?

15. Why was shotgun metagenomic sequencing not performed to validate primer specificity and assess overall merA diversity?

16. On what basis do you generalize horizontal gene transfer potential from sequencing a single Citrobacter freundii isolate?

17. Why were plasmid conjugation or transfer experiments not conducted to experimentally confirm horizontal transfer capability?

18. How might environmental factors (e.g., pH, sediment mercury, proximity to mining, industrial discharge) influence merA prevalence, and why were these not assessed?

19. Did you analyze potential co-selection between mercury resistance genes and antibiotic resistance genes in your plasmid or metagenomic data?

20. Given the absence of direct human mercury exposure measurements, how do you justify the conclusion that fecal merA analysis can serve as a proxy for mercury-related public health risk?

21.Provide the proper citation in material and methods- Add below appropriate reference-

Nazaret, S., Jeffrey, W.H., Saouter, E., Von Haven, R. and Barkay, T., 1994. merA gene expression in aquatic environments measured by mRNA production and Hg (II) volatilization. Applied and Environmental Microbiology, 60(11), pp.4059-4065.

Gendy, S., Pathak, A., Agarwal, M., Rathore, R.S. and Chauhan, A., 2020. Draft genome sequence of mercury-resistant Serratia sp. strain SRS-8-S-2018. Microbiology Resource Announcements, 9(15), pp.10-1128.

Rothenberg, S.E., Sweitzer, D.N., Rackerby, B.R., Couch, C.E., Cohen, L.A., Broughton, H.M., Steingass, S.M. and Beechler, B.R., 2021. Fecal methylmercury correlates with gut microbiota taxa in Pacific walruses (Odobenus rosmarus divergens). Frontiers in Microbiology, 12, p.648685.

Agarwal, M., Rathore, R.S., Jagoe, C. and Chauhan, A., 2019. Multiple lines of evidences reveal mechanisms underpinning mercury resistance and volatilization by Stenotrophomonas sp. MA5 isolated from the Savannah River Site (SRS), USA. Cells, 8(4), p.309.

Gaeta, N.C., de Carvalho, D.U., Fontana, H., Sano, E., Moura, Q., Fuga, B., Munoz, P.M., Gregory, L. and Lincopan, N., 2022. Genomic features of a multidrug-resistant and mercury-tolerant environmental Escherichia coli recovered after a mining dam disaster in South America. Science of the Total Environment, 823, p.153590.

Pathak, A., Agarwal, M., Rathore, R.S. and Chauhan, A., 2019. Gene determinants for mercury bioremediation as revealed by draft genome sequence analysis of Stenotrophomonas sp. strain MA5. Microbiology Resource Announcements, 8(17), pp.10-1128.

[Note: HTML markup is below. Please do not edit.]

Reviewers' comments:

Reviewer's Responses to Questions

**Comments to the Author**

Reviewer #4: (No Response)

**********

2. Is the manuscript technically sound, and do the data support the conclusions?

Reviewer #4: No

**********

3. Has the statistical analysis been performed appropriately and rigorously?

Reviewer #4: No

**********

4. Have the authors made all data underlying the findings in their manuscript fully available?

The PLOS Data policy

Reviewer #4: No

**********

5. Is the manuscript presented in an intelligible fashion and written in standard English?

Reviewer #4: No

**********

Reviewer #4: The manuscript entitled “Multinational comparison of the prevalence of the mercury resistance biomarker gene merA in human feces, food, and environmental water” aims to investigate the abundance of the merA gene across different sample types collected from multiple countries. The topic is relevant and potentially impactful; however, the manuscript can be accepted in its current form. It requires substantial revision before it can be considered for further review. Some of the concerns are below:

-The use of the term “Multinational” in the title is misleading, as the study includes samples from only three countries. The title should be revised to more accurately reflect the geographic scope of the investigation

-The short title suggests that merA is being proposed as a biomarker for mercury contamination. However, merA is already well established as a central gene in the mer operon, and this study does not demonstrate novelty in that regard. The title and conclusions should instead emphasize screening, distribution, or comparative prevalence of merA across different matrices and regions.

-The manuscript contains repetitive and inconsistent phrasing (e.g., “Mercury (Hg) is a widespread environmental pollutant” followed by “Mercury is a toxic environmental pollutant”. Redundancy should be avoided. Abbreviations should be used consistently. Once introduced the abbreviation should be used throughout the manuscript.

- In the introduction section, The authors state that methylmercury is the most toxic form of mercury affecting humans; however, the study focuses exclusively on merA, which is involved in the reduction of Hg(II) and does not play a role in methylmercury detoxification. The introduction is poorly written and lacks logical flow. No clear detoxification pathway is described, and the role of merA within the mer operon, including its functional importance, is not adequately explained.

-The manuscript does not identify the original sources of mercury contamination in the three countries studied.

-Information on environmental mercury levels in these regions (water, soil, food chains) should be provided to support the interpretation of merA prevalence.

-The overall study design is vague. The rationale for selecting specific sample sources (human feces, food, environmental water) is not clearly explained. It is unclear whether the sampled populations were exposed to mercury through environmental contamination, dietary intake, proximity to polluted water bodies, or other sources. For environmental water samples, the authors should clarify whether samples were filtered prior to analysis and whether turbidity, dust, or sand particles were present.

-The basis for selecting 60 μM HgCl^2^ for bacterial isolation is not provided. The authors should justify this concentration with references or preliminary data.

-The source of Citrobacter freundii strain TB25-009W-60-9 is unclear.

-Given the extensive literature on the mer operon, the rationale for performing additional isolation and genomic analysis of mercury-resistant strains should be better justified.

-Several subsection headings in the Results section resemble those of the Materials and Methods section and should be revised to reflect actual findings rather than procedures.

-The authors should clarify whether the mercury detected in human fecal samples is more likely linked to dietary sources (e.g., chicken consumption) or drinking water contamination.

- I do not find the figures in this version of ms.

-The isolation of mercury-resistant bacteria and genomic analysis of a single strain are not well aligned with the stated objectives in the Introduction.

-The authors should either strengthen the rationale linking these analyses to the study goals or revise the scope of the manuscript accordingly.

**********

what does this mean?). If published, this will include your full peer review and any attached files.

If you choose “no”, your identity will remain anonymous but your review may still be made public.

**Do you want your identity to be public for this peer review?**  For information about this choice, including consent withdrawal, please see our Privacy Policy

Reviewer #4: No

**********

[NOTE: If reviewer comments were submitted as an attachment file, they will be attached to this email and accessible via the submission site. Please log into your account, locate the manuscript record, and check for the action link "View Attachments". If this link does not appear, there are no attachment files.]

To ensure your figures meet our technical requirements, please review our figure guidelines: https://journals.plos.org/plosone/s/figures

You may also use PLOS’s free figure tool, NAAS, to help you prepare publication quality figures: https://journals.plos.org/plosone/s/figures#loc-tools-for-figure-preparation.

NAAS will assess whether your figures meet our technical requirements by comparing each figure against our figure specifications.

# Author comment: Author response to Decision Letter 3

**DOI:** 10.1371/journal.pone.0357976.r006

17 Mar 2026

We sincerely thank the Editor and Reviewer for this careful evaluation of our manuscript and for the constructive and detailed comments, which have helped improve the clarity of our study. We have revised the manuscript extensively in response to all points raised.

Attachment Submitted filename: Response to reviewers.docx

# Peer review: Decision Letter 3

**DOI:** 10.1371/journal.pone.0357976.r007

**Authors:** Xin Sun

7 Jul 2026

Dear Dr. Yamamoto,

Thank you for submitting your manuscript to PLOS ONE. After careful consideration, we feel that it has merit but does not fully meet PLOS ONE’s publication criteria as it currently stands. Therefore, we invite you to submit a revised version of the manuscript that addresses the points raised during the review process.

Please submit your revised manuscript by Aug 21 2026 11:59PM. If you will need more time than this to complete your revisions, please reply to this message or contact the journal office at plosone@plos.org. When you're ready to submit your revision, log on to https://www.editorialmanager.com/pone/ and select the 'Submissions Needing Revision' folder to locate your manuscript file.

- A letter that responds to each point raised by the academic editor and reviewer(s). You should upload this letter as a separate file labeled 'Response to Reviewers'.
- A marked-up copy of your manuscript that highlights changes made to the original version. You should upload this as a separate file labeled 'Revised Manuscript with Track Changes'.
- An unmarked version of your revised paper without tracked changes. You should upload this as a separate file labeled 'Manuscript'.

If you would like to make changes to your financial disclosure, please include your updated statement in your cover letter. Guidelines for resubmitting your figure files are available below the reviewer comments at the end of this letter.

If applicable, we recommend that you deposit your laboratory protocols in protocols.io to enhance the reproducibility of your results. Protocols.io assigns your protocol its own identifier (DOI) so that it can be cited independently in the future. For instructions see: https://journals.plos.org/plosone/s/submission-guidelines#loc-laboratory-protocols. Additionally, PLOS ONE offers an option for publishing peer-reviewed Lab Protocol articles, which describe protocols hosted on protocols.io. Read more information on sharing protocols at https://plos.org/protocols?utm_medium=editorial-email&utm_source=authorletters&utm_campaign=protocols.

As the corresponding author, your ORCID iD is verified in the submission system and will appear in the published article. PLOS supports the use of ORCID, and we encourage all coauthors to register for an ORCID iD and use it as well. Please encourage your coauthors to verify their ORCID iD within the submission system before final acceptance, as unverified ORCID iDs will not appear in the published article. *Only*  the individual author can complete the verification step; PLOS staff *cannot*  verify ORCID iDs on behalf of authors.

We look forward to receiving your revised manuscript.

Kind regards,

Xin Sun, PhD

Staff Editor

PLOS One

Journal Requirements:

If the reviewer comments include a recommendation to cite specific previously published works, please review and evaluate these publications to determine whether they are relevant and should be cited. There is no requirement to cite these works unless the editor has indicated otherwise.

Please review your reference list to ensure that it is complete and correct. If you have cited papers that have been retracted, please include the rationale for doing so in the manuscript text, or remove these references and replace them with relevant current references. Any changes to the reference list should be mentioned in the rebuttal letter that accompanies your revised manuscript. If you need to cite a retracted article, indicate the article’s retracted status in the References list and also include a citation and full reference for the retraction notice.

[Note: HTML markup is below. Please do not edit.]

Reviewers' comments:

Reviewer's Responses to Questions

**Comments to the Author**

Reviewer #5: (No Response)

**********

2. Is the manuscript technically sound, and do the data support the conclusions?

Reviewer #5: Yes

**********

3. Has the statistical analysis been performed appropriately and rigorously?

Reviewer #5: Yes

**********

4. Have the authors made all data underlying the findings in their manuscript fully available?

The PLOS Data policy

Reviewer #5: Yes

**********

5. Is the manuscript presented in an intelligible fashion and written in standard English?

Reviewer #5: Yes

**********

Reviewer #5: This study demonstrates the prevalence of the mercury detoxification gene (merA) in human stool, chicken meat, and water samples from three different countries. Mercury contamination is a significant problem, and this study highlights the implications of mercury released from anthropogenic sources by detecting one of the genetic biomarkers of mercury detoxification. I have attached a few comments and questions below:

Line 56 in the introduction is redundant and may be removed as the same information is conveyed in other sentences. Hg(II) is never mentioned clearly as the most common and/or toxic form of environmental mercury, which may be important as the study revolves around merA, an Hg(II) reducing gene. Line 72 may include a sentence about any previous literature indicating the prevalence of HGT of mercury resistance or even heavy metal resistance genes in aquatic ecosystems, since it is relevant to the genome analysis performed in the study.

In line 145 of the methods section, it is more meaningful to include the concentration rather than the volume of DNA obtained.

In the results for the abundance of merA, the quantification results would have benefited from the use of a “control” gene, such as the 16S, in order to properly assess the microbial population abundance in the samples. The authors have mentioned that they extracted DNA from the same amount of starting material, but were the final DNA concentrations comparable between samples after extraction, or was any kind of normalization performed between samples for better interpretation of merA abundance?

The lack of mercury measurements from the stool and meat samples, which will help correlate the Hg prevalence and merA abundance, is never addressed, especially since mercury is bioaccumulated across the trophic levels.

Were any sequence similarity or phylogenetic analyses performed for the detected IncFIB plasmid to infer the occurrence of an HGT event?

**********

what does this mean?). If published, this will include your full peer review and any attached files.

If you choose “no”, your identity will remain anonymous but your review may still be made public.

**Do you want your identity to be public for this peer review?**  For information about this choice, including consent withdrawal, please see our Privacy Policy

Reviewer #5: No

**********

[NOTE: If reviewer comments were submitted as an attachment file, they will be attached to this email and accessible via the submission site. Please log into your account, locate the manuscript record, and check for the action link "View Attachments". If this link does not appear, there are no attachment files.]

To ensure your figures meet our technical requirements, please review our figure guidelines: https://journals.plos.org/plosone/s/figures

You may also use PLOS’s free figure tool, NAAS, to help you prepare publication quality figures: https://journals.plos.org/plosone/s/figures#loc-tools-for-figure-preparation.

NAAS will assess whether your figures meet our technical requirements by comparing each figure against our figure specifications.

# Author comment: Author response to Decision Letter 4

**DOI:** 10.1371/journal.pone.0357976.r008

21 Jul 2026

PONE-D-25-30013R3

Manuscript title: “Comparative prevalence of the mercury resistance gene merA in human feces, food, and environmental water from Japan, Vietnam, and Ghana”.

We sincerely thank Reviewer for your through and this careful evaluation of our manuscript. Your insightful comments and questions have helped us significantly improve the clarity of our work. We have revised the manuscript extensively in response to all suggestions.

Below we provide a point-by-point response. All changes have been incorporated into the revised manuscript and are indicated accordingly.

Response to Reviewer #5

Comment 1:

Line 56 in the introduction is redundant and may be removed as the same information is conveyed in other sentences. Hg(II) is never mentioned clearly as the most common and/or toxic form of environmental mercury, which may be important as the study revolves around merA, an Hg(II) reducing gene.

►Response:

We thank to Reviewer for the valuable observation. We have removed the redundant sentence from line 56 and added a brief explanation of Hg(II)(line 56-63), as suggested.

Comment 2:

Line 72 may include a sentence about any previous literature indicating the prevalence of HGT of mercury resistance or even heavy metal resistance genes in aquatic ecosystems, since it is relevant to the genome analysis performed in the study.

►Response:

We thank to Reviewer for this constructive suggestion. We have added a sentence in the Introduction section (line 83-86) referencing previous studies in horizontal gene transfer of mercury resistance genes in aquatic environments which provide context for our genomic analysis of the mer operon localization plasmid.

Comment 3:

In the line 145 of the methods section, it is more meaningful to include the concentration rather than the volume of DNA obtained. In the results for the abundance of merA, the quantification results would have benefited from the use of a “control” gene, such as the 16S, in order to properly assess the microbial population abundance in the samples. The authors have mentioned that they extracted DNA from the same amount of starting material, but were the final DNA concentrations comparable between samples after extraction, or was any kind of normalization performed between samples for better interpretation of merA abundance?

►Response:

We thank to Reviewer for identifying these important points.

Regarding normalization and the use of a control gene, we appreciate this valuable methodological suggestion. We agree that normalization to a housekeeping gene such as the 16S rRNA gene would provide a more refined assessment of merA abundance relative to the total bacterial load. However, our primary aim was to compare merA detection rates and relative copy numbers under consistent experimental conditions rather than to determine absolute merA abundance. In accordance with the reviewer's suggestion, we have added the range of DNA concentrations obtained from the extracted samples to the Materials and methods section (line 168-171). We have also revised the Discussion to include a description of the limitations of this method (line 366-375).

Comment 4:

The lack of mercury measurements from the stool and meat samples, which will help correlate the Hg prevalence and merA abundance, is never addressed, especially since mercury is bioaccumulated across the trophic levels.

Were any sequence similarity or phylogenetic analyses performed for the detected IncFIB plasmid to infer the occurrence of an HGT event?

►Response:

We thank to Reviewer for the insightful comment.

Regarding mercury measurements in stool and meat samples, the measuring total mercury concentrations in stool and meat samples would have provided valuable complementary data to correlate with merA abundance. However, the primary objective of this study was to investigate the prevalence of the merA gene as a biomarker of mercury resistance, rather than to directly quantify mercury levels in biological matrices. While mercury bioaccumulation across trophic levels is well documented, the relationship between mercury concentrations and merA prevalence is complex, as merA reflects the presence of mercury-resistant bacteria rather than directly measuring mercury content. We have added a discussion of this limitation and the need for future studies incorporating direct mercury measurements in the biological samples to the Limitations section (line 408-420).

Regarding phylogenetic analysis of the IncFIB plasmid, we performed comparative genomic analysis of the IncFIB plasmid from C. freundii TB25-009W-60-9 against the reference plasmid from Klebsiella pneumonia 1_GR_13. The analysis revealed that mer operon structure was nearly identical between the two plasmids, while most other genetic regions showed no homology (Fig S2). This finding suggests that mer operon may have been acquired by the IncFIB plasmid through HGT events. Additionally, the mer operon in our isolate was flanked by two insertion sequences (ISs), which further supports the potential for horizontal transfer. We have expanded the Discussion section (line 384-391) to include these observations and their implication for HGT.

Attachment Submitted filename: Response_to_reviewers_auresp_4.docx

# Peer review: Decision Letter 4

**DOI:** 10.1371/journal.pone.0357976.r009

**Authors:** Ali Hasan

26 Aug 2026

Comparative prevalence of the mercury resistance gene merA in human feces, food, and environmental water from Japan, Vietnam, and Ghana

PONE-D-25-30013R4

Dear Dr. Yamamoto,

We’re pleased to inform you that your manuscript has been judged scientifically suitable for publication and will be formally accepted for publication once it meets all outstanding technical requirements.

Within one week, you’ll receive an e-mail detailing the required amendments. When these have been addressed, you’ll receive a formal acceptance letter and your manuscript will be scheduled for publication.

An invoice will be generated when your article is formally accepted. Please note, if your institution has a publishing partnership with PLOS and your article meets the relevant criteria, all or part of your publication costs will be covered. Please make sure your user information is up-to-date by logging into Editorial Manager at Editorial Manager® and clicking the ‘Update My Information' link at the top of the page. For questions related to billing, please contact billing support.

If your institution or institutions have a press office, please notify them about your upcoming paper to help maximize its impact. If they’ll be preparing press materials, please inform our press team as soon as possible -- no later than 48 hours after receiving the formal acceptance. Your manuscript will remain under strict press embargo until 2 pm Eastern Time on the date of publication. For more information, please contact onepress@plos.org.

Kind regards,

Ali Hasan, PhD

Academic Editor

PLOS One

Additional Editor Comments (optional):

Thank you for submitting your manuscript to Plos One. Based on the reviewer's comments, I am pleased to inform you that your manuscript has been accepted for publication.

Reviewers' comments:

Reviewer's Responses to Questions

**Comments to the Author**

Reviewer #5: All comments have been addressed

**********

2. Is the manuscript technically sound, and do the data support the conclusions?

Reviewer #5: Yes

**********

3. Has the statistical analysis been performed appropriately and rigorously?

Reviewer #5: Yes

**********

4. Have the authors made all data underlying the findings in their manuscript fully available?

The PLOS Data policy

Reviewer #5: Yes

**********

5. Is the manuscript presented in an intelligible fashion and written in standard English?

Reviewer #5: Yes

**********

Reviewer #5: The authors have addressed previous comments satisfactorily. I have no additional suggestions for the manuscript.

**********

what does this mean?). If published, this will include your full peer review and any attached files.

If you choose “no”, your identity will remain anonymous but your review may still be made public.

**Do you want your identity to be public for this peer review?**  For information about this choice, including consent withdrawal, please see our Privacy Policy

Reviewer #5: No

**********

# Editor report: Acceptance letter

**DOI:** 10.1371/journal.pone.0357976.r010

**Authors:** Ali Hasan

PONE-D-25-30013R4

PLOS One

Dear Dr. Yamamoto,

I'm pleased to inform you that your manuscript has been deemed suitable for publication in PLOS One. Congratulations! Your manuscript is now being handed over to our production team.

At this stage, our production department will prepare your paper for publication. This includes ensuring the following:

* All references, tables, and figures are properly cited

* All relevant supporting information is included in the manuscript submission,

* There are no issues that prevent the paper from being properly typeset

You will receive further instructions from the production team, including instructions on how to review your proof when it is ready. Please keep in mind that we are working through a large volume of accepted articles, so please give us a few days to review your paper and let you know the next and final steps.

Lastly, if your institution or institutions have a press office, please let them know about your upcoming paper now to help maximize its impact. If they'll be preparing press materials, please inform our press team within the next 48 hours. Your manuscript will remain under strict press embargo until 2 pm Eastern Time on the date of publication. For more information, please contact onepress@plos.org.

You will receive an invoice from PLOS for your publication fee after your manuscript has reached the completed accept phase. If you receive an email requesting payment before acceptance or for any other service, this may be a phishing scheme. Learn how to identify phishing emails and protect your accounts at https://explore.plos.org/phishing.

If we can help with anything else, please email us at customercare@plos.org.

Thank you for submitting your work to PLOS One and supporting open access.

Kind regards,

PLOS One Editorial Office Staff

on behalf of

Dr. Ali Hasan

Academic Editor

PLOS One
